# A tutorial for software options to aid in assessing functional relations in single-case experimental designs

**DOI:** 10.3758/s13428-026-02951-z

**Published:** 2026-02-23

**Authors:** Rumen Manolov

**Affiliations:** Department of Social Psychology and Quantitative Psychology, Faculty of Psychology, University of Barcelona, Passeig de la Vall d’Hebron 171, 08035 Barcelona, Spain

**Keywords:** Single-case experimental designs, Functional relation, Visual analysis, Software

## Abstract

Single-case experimental designs (SCEDs) can be used for identifying effective interventions via the intensive study of one or a few individuals in different conditions, actively manipulated by the researcher. The assessment of SCED data entails both judging whether there is sufficient evidence of a functional relation (i.e., a causal effect of the intervention on the target behavior) and quantifying the magnitude of the effect. In the current text, the focus is on assessing the presence of a functional relation, considering all the attempts to demonstrate an effect that SCEDs include. Specifically, the aim is to review several freely available websites, which require no additional software to be installed, and offer graphical representations of the data, visual aids, and quantifications. Several data analytical steps are outlined for performing the assessment, both dealing with each basic effect separately and evaluating the consistency of effects. Software that is useful for carrying out these steps is reviewed, including the way in which the data files should be specified and the few clicks required by applied researchers to achieve the desired output. The interpretations of the output are illustrated with real data.

Single-case experimental designs (SCEDs) offer a valid means of providing evidence regarding the effect of interventions (Horner et al., 2005; Jenson et al., 2007; Schlosser, 2009), by meeting certain methodological requirements (Ganz & Ayres, 2018; Perdices et al., 2023; Tanious et al., 2024). One of these requirements is having several attempts to show an intervention effect, that is, several changes between conditions at different moments in time, either for the same unit or for different units (Perdices et al., 2023; What Works Clearinghouse, 2022). This can be understood as a within-study replication of the intervention effect, which can be performed within the same participant (e.g., in a reversal design, alternating-treatment design, or multiple-baseline design across settings or behaviors), or it may require having several participants available (e.g., in a multiple-baseline design across participants). It is common to require at least three attempts to demonstrate an intervention effect in order to consider a design methodologically strong (Tate et al., 2013; What Works Clearinghouse, 2022; but see Cariveau & Lewis, 2025).

Within-study replication enhances internal validity (Morley, 2018; Tate & Perdices, 2019), as the main aim of a single study is to provide evidence regarding a functional (causal) relation between the intervention and the changes in the outcome. This could also be referred to as “simultaneous replication,” to be distinguished from “sequential replication” more focused on generalizability (Michiels & Onghena, 2019). Similar to the latter, the main aim of a meta-analytical integration of several studies (i.e., cross-study replication) is to provide an overall quantification of the intervention effect and an assessment of the generality of the effect or external validity.

Regarding the assessment of intervention effectiveness in the SCED context, it can be performed from several complementary perspectives. While data collection is still ongoing, a formative analysis can be performed by continually plotting the measurements being obtained and making any necessary adaptations in the intervention or decisions regarding the change in phases (Barton et al., 2016). In contrast, a summative analysis is performed once data collection is over, and the aim is to document the intervention effect. For a formative analysis and for deciding whether there a functional relation is present (as an instance of summative analysis), visual analysis is important alongside considering the features of the design (Ledford et al., 2019; Kratochwill et al., 2021, 2023). The identification of the presence of an effect can be complemented by performing another kind of summative analysis, using quantifications of the magnitude of effect, which bring objectivity and the possibility of a quantitative research synthesis (Kratochwill et al., 2023; Pustejovsky & Ferron, 2017). Recent studies have also proposed a meta-analytical integration complemented by visual representations of the data (Kinney et al., 2025; Tanious & Manolov, 2022). Specifically, the proposal by Kinney and colleagues (2025) emphasizes the importance of not only computing an effect size when performing a meta-analysis but also using “structured criteria [in each study] to visually analyze the generated graphs and verify whether a functional relation had been demonstrated between the intervention and the dependent variable” (p. 348). Complementarily, also in the context of the quantitative integration of the results of several studies, Tanious and Manolov (2022) propose representing all data points via violin plots (one for the baseline condition and another for the intervention condition) in order to allow for comparisons beyond summary measures such as means.

Finally, beyond the use of visual analysis for identifying the presence of a functional relation and quantifying the magnitude of the effect, it is important to assess social validity. This assessment includes taking into account the importance of the target outcome for the individual, the acceptability and feasibility of the intervention, and the real-life impact and maintenance of the intervention effect (Kazdin, 1977; Snodgrass et al., 2023). As part of the evaluation of social validity, it can be considered whether and to what extent a pre-established goal (e.g., a mastery criterion) has been achieved (Ferron et al., 2020). A mastery criterion can be understood as a level of behavior that enables normal everyday life functioning, for instance, in comparison to typically developing peers. Thus, such a level would transcend labels such as “statistically significant” or “large” and would translate to a meaningful effect for the individual’s activities and/or well-being, outside the context of the research.

## Aims, scope, and organization of this paper

The aim of the current text is to present how different freely accessible web-based applications can be used by applied researchers for the assessment of functional relations in SCED data. A structured approach to visual analysis is necessary (see proposals by Maggin et al., 2013; Wolfe et al., 2019) in order to overcome the insufficient interrater agreement (Bishara et al., 2021; Ninci et al., 2015; Wilbert et al., 2021). Moreover, it is crucial to improve reporting of how visual analysis is performed (Wolfe et al., 2024). A tutorial on software tools making possible a systematic visual analysis is a relevant step in this direction. The current tutorial is also necessary because, as it will be illustrated, there are multiple tools that can aid in the assessment of functional relations, scattered over several websites, each with its own characteristics.

It is important to note that the initial version of the What Works Clearinghouse standards (Kratochwill et al., 2010) clearly separated the assessment of the presence of a functional relation via visual analysis from the quantification of effects, with the latest version (What Works Clearinghouse, 2022) focusing only on the latter. Nonetheless, there were reactions against the omission of visual analysis (Kratochwill et al., 2021; Maggin et al., 2022) along with a recent emphasis on functional relations (Gilroy et al., 2025). For these reasons, the current text focuses on functional relations.

As it is beyond the scope of the current text, the reader interested in quantifying effects can consult several different sources, written as tutorials, as follows: (a) for the between-case standardized mean difference (Shadish et al., 2014), Valentine et al. (2016); (b) for the implementation of multilevel models (Ferron et al., 2009, 2010), Declercq et al. (2020) and Manolov and Moeyaert (2025); see also Li et al. (2025); (c) for randomization tests (Heyvaert & Onghena, 2014; Levin & Kratochwill, 2021), Bulté and Onghena (2013) and Levin et al. (2014); and (d) for Bayesian analysis, Natesan (2019) and Natesan Batley (2023).

Given that the assessment of overlap is usually part of visual analysis, in the current text we will refer to software implementations making possible the quantification and graphical representation of overlap. This is also well aligned with the idea that nonoverlap indices (see Parker, Vannest, & Davis, 2011a, 2011b, for a review, and for later proposals see Tarlow, 2017; Manolov & Tanious, 2025) may not be understood as “effect sizes” in the same way as a standardized mean difference or a log response ratio due to the presence of a ceiling effect (i.e., no quantification of distance once complete nonoverlap is achieved; Carter, 2013; Wolery et al., 2010).

Regarding the organization of the paper, first we provide a brief review of the assessment of functional relations. Second, we present the data that we will use for illustrating the software implementations reviewed here. Third, we refer to the software that will be useful for several data analytical steps and the required formats for the data files. Fourth, we present the results of the use of the websites for the illustrative data. Finally, we provide a discussion, referring to topics open to debate and to further readings on software for SCED data analysis.

## Functional relations

To begin with, it should be noted that in certain situations, the two kinds of SCED data analysis (assessing functional relations and quantifying the magnitude of effect) may not coincide completely. For instance, in the context of a multiple-baseline design, the between-case standardized mean difference (Hedges et al., 2013) and multilevel models (Ferron et al., 2009) quantify the difference between the adjacent baseline and intervention phases within each tier, but pay no attention to the staggered introduction of the intervention unless a specific model is used (Ferron et al., 2014). Similarly, for reversal (ABAB) designs, the between-case standardized mean difference (Hedges et al., 2012) omits the comparison between the first intervention and the second baseline phase from the quantification, which may not be methodologically desirable (Tanious & Manolov, 2025). Specifically, in the case that there is a complete reversal in A_2_ relative to A_1_ (and thus a large difference between B_1_ and A_2_), omitting this information may underestimate the effect. Contrarily, in the case of a small or no reversal (apart from being a problem for the functional relation), omitting this small or null effect from the quantification may overestimate the effect. In contrast to the between-case standardized mean difference, when using a multilevel model, the researcher can select the coding in order to include or exclude this comparison (see Shadish et al., 2013).

Regarding the use of visual analysis for assessing functional relations (see Kratochwill et al., 2010; Lane & Gast, 2014; Ledford et al., 2019; Maggin et al., 2018), an initial step consists of focusing on within-phase data patterns in order to check whether there is a clear (predictable) pattern within each condition. Given that level, trend, and variability are assessed, the stability around a mean or a trend line is especially important (Lane & Gast, 2014). Subsequently, adjacent phases (baseline and intervention) are compared, with each two-phase comparison being considered a basic effect (Horner & Odom, 2014) and requiring at least three such basic effects to be present (What Works Clearinghouse, 2022). Specifically, it is possible to identify changes in level, trend, or variability, as well as the amount of overlap between measurements belonging to different conditions. Moreover, it is possible to assess whether the effect is immediate or not. Overall, the assessment performed via visual analysis can be understood as comparing the projection of the baseline data with the actual intervention phase measurements (Kratochwil et al., 2010). Several proposals reflect this latter idea (e.g., Fisher et al., 2003; Manolov & Vannest, 2023; Pfadt & Wheeler, 1995).

In relation to the five previously mentioned data features (level, trend, variability, overlap, and immediacy), it is important to determine, before data collection, which data feature is the focus of the analysis, according to the expectation (Manolov et al., 2022a, 2022b), as is common in the context of randomization tests (Heyvaert & Onghena, 2014; Levin et al., 2017, 2021). Such a practice is also well aligned with the recent calls for transparency (including preregistration; Cook et al., 2022; Johnson & Cook, 2019; Tincani et al., 2024) and the need to avoid questionable research practices (Tincani et al., 2025), potentially leading to confirmation bias (Laraway et al., 2019).

Once a focal data feature is chosen, it is important to consider, across all comparisons between a baseline and an intervention condition, whether there is enough evidence of an intervention effect. This entails considering all basic effects together and assessing their consistency (Kratochwill et al., 2010; Lane et al., 2017; Ledford, 2018; see also Tanious et al., 2020, 2021). Here we suggest following the logic of a “success rate” (Reichow & Volkmar, 2010; see also Hagopian, 2020), which is essentially equivalent to Speelman and McGann’s (2020) proposal to assess the “pervasiveness” of an effect as the proportion of people (here, A–B comparisons^1^) for whom a desirable effect is observed. Specifically, one proposal from the SCED context is the requirement for a ratio of at least 3:1 positive (desirable) effects for each null effect (Cook et al., 2015; Maggin et al., 2013), although each researcher can judge whether this ratio is sufficient for considering that the intervention is effective.

On the one hand, a positive effect can be understood as the one for which the difference is of the expected sign (Maggin et al., 2011), that is, the intervention is better than the baseline. On the other hand, it is possible to define a minimally relevant effect in relation to the importance of social validity. Apart from inspecting the typical time-series plots, the assessment of consistency can also benefit from the use of the modified Brinley plot (Blampied, 2017), in which the baseline and intervention phase means^2^ for several basic effects can be represented jointly, and it is possible to assess whether the sign and magnitude of the difference in means is consistent (Manolov & Tanious, 2022; Manolov et al., 2022a, 2022b).

## Context

**Fig. 1:**
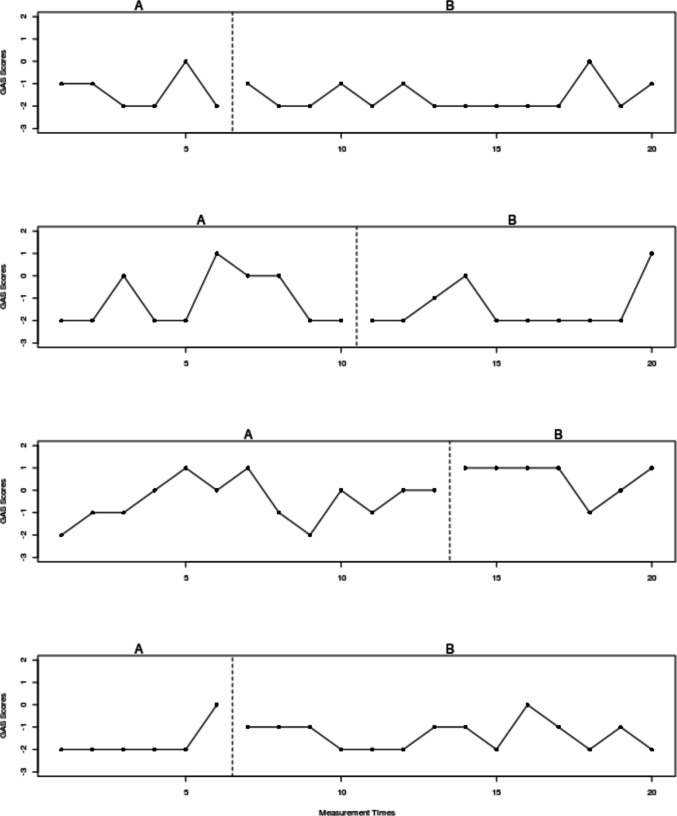
Multiple-baseline data for Ian, representing three treated behaviors and a control behavior. *Note*. Graph obtained via https://tamalkd.shinyapps.io/scda/, using a datafile structured as depicted in Fig. 5. **A** represents baseline; **B** represents intervention phase

The current example is based on the data^3^ gathered by Lebrault et al. (2024), studying the effect of an intervention called “Cognitive Orientation to daily Occupational Performance” on occupational performance goals for children with executive function deficits after acquired brain injury. The authors report three multiple-baseline studies (an initial study and two replications), each with four participants and four goals per participant. The fourth goal for each participant was an untrained control goal not expected to change unless transfer occurred. The attainment of the different goals was measured via a Goal Attainment Scale, which is an ordinal measure with six possible values, following Steenbeek et al. (2010): −3, performance below the initial level; −2, initial level; −1, less improvement than expected; 0, expected goal; +1, somewhat more than expected; and +2, much more than expected. In the current illustration, we use the data for “Replication 2” for participant Ian (see Fig. 1).

This dataset was chosen because it represents the most commonly used type of SCED (per reviews by Ledford et al., 2024; Shadish & Sullivan, 2011; Tanious & Onghena, 2021): the multiple-baseline design (Carr, 2005; Slocum et al., 2022). Moreover, the measurement scale of the main dependent variable is ordinal, which presents an analytical challenge to applied researchers that is worth noting. A quantification based on distances (e.g., a mean difference) would be less appropriate, as it requires the assumption of constant distance (i.e., a dependent variable measured in an interval or ratio scale). In contrast, a quantification of nonoverlap—one of the six visually assessed data features—only entails ordinal comparisons (i.e., which value is smaller and which value is larger, but not how much smaller or larger). Finally, out of all participants studied, participant Ian’s data (Fig. 1) show certain variability that makes the analysis more challenging. For illustrative purposes and for the assessment of consistency of effects, we will assume that 0 is the minimum desirable value for the intervention phase, as it represents the expected goal. We will also assume that a difference of at least 1 point on average is required for the intervention to be considered effective.

## Software implementations reviewed

### Data analysis plan and links to the websites

The following steps will be carried out:

1. For each basic effect (i.e., each A–B comparison), assess the within-phase pattern, focusing on data variability in order to gauge whether the data show a predictable level or trend. It is possible to use any website (e.g., https://tamalkd.shinyapps.io/scda; https://jepusto.shinyapps.io/SCD-effect-sizes) or an Excel macro (https://ex-prt.weebly.com/) or Excel templates (https://osf.io/bt9hf/), allowing us to construct a time-series plot; https://manolov.shinyapps.io/Overlap/, for instance, includes a stability envelope (Lane & Gast, 2014).
2. Again, for each basic effect, assess the focal data feature (level, trend, variability, overlap, immediacy) chosen according to a priori expectations. For the current illustration, we will suppose that variable data are expected, but no spontaneous improvement (i.e., problematic baseline trend). Therefore, summarizing the data by a mean or a trend line may not be reasonable in the case of lack of (trend) stability. We will focus on overlap and choose one specific operational definition: the one entailed in the Nonoverlap of All Pairs (NAP; Parker & Vannest, 2009), in which all baseline measurements are compared to all intervention phase measurements, obtaining a quantification equivalent to a probability of superiority (Grissom & Kim, 2001).
  - For level, consider the mean or the median, according to the measurement scale and the presence of potential outlying values. https://manolov.shinyapps.io/Overlap/ and http://www.interventioncentral.org/teacher-resources/graph-maker-free-onlinecan be used. The median can also be represented via https://ansa.shinyapps.io/ansa/.
  - For trend, consider different possible trend line fitting techniques. https://manolov.shinyapps.io/TrendMASE can be used. Further quantifications, specifically based on regression, can be obtained via several websites (https://manolov.shinyapps.io/Regression/, https://mirisola-unipa.shinyapps.io/unipa-scr-dev/, and http://www.interventioncentral.org/teacher-resources/graph-maker-free-online) and an R package (https://cran.r-project.org/web/packages/scan/index.html). Additionally, for monotonic trend, https://ansa.shinyapps.io/ansa/ can be used.
  - For projecting level and trend, compare the results of the conservative dual criteria projecting the mean level and the split-middle trend (Fisher et al., 2003) with the projection of the Theil–Sen trend line (see Vannest et al., 2012, for an application to SCED data) considering the variability of the baseline data (Manolov & Vannest, 2023). For the former, use https://manolov.shinyapps.io/Overlap/; for the latter, use https://manolov.shinyapps.io/TrendMAD. The *p* value corresponding to the conservative dual criterion can be obtained via a binomial test (e.g., https://www.graphpad.com/quickcalcs/binomial1/), quantifying the probability of as many or more points from the intervention phase that fall above or below (according to the effect desired) the projected baseline split-middle trend.
  - For immediacy,^4^ assess the difference between the final three baseline measurements and the initial three intervention phase measurements (Horner & Kratochwill, 2012). Following the logic of sensitivity analysis, assess immediacy in a different way as well: on the basis of the projection of the baseline trend to the first intervention phase measurement occasion, as defined in piecewise regression (Center et al., 1985; Moeyaert, Ugille, et al., 2014a, 2014b). Both options are implemented at https://manolov.shinyapps.io/Overlap/, with piecewise regression quantifications also available at https://manolov.shinyapps.io/Regression/.
  - For overlap, consider whether baseline trend has to be taken into account or not (Manolov & Tanious, 2025; Parker, Vannest, Davis, & Sauber, 2011a, 2011b; Tarlow, 2017). Assess the amount of overlap and whether a ceiling effect is present. Multiple websites (e.g., https://singlecaseresearch.org/; https://ktarlow.com/stats/; https://jepusto.shinyapps.io/SCD-effect-sizes; http://www.interventioncentral.org/teacher-resources/graph-maker-free-online ) and R packages (e.g., https://cran.r-project.org/web/packages/scan/index.html) implement nonoverlap indices, but here we focus on the website that serves multiple purposes related to visual analysis (https://manolov.shinyapps.io/Overlap/).
3. For all basic effects considered together, for assessing consistency:
  - Compare the average baseline and intervention trend lines and the average intervention effects with the trends and effects for each tier (here, behavior for Ian). This can be achieved by visual inspection via the two-level model implemented in https://manolov.shinyapps.io/Overlap/, http://34.251.13.245/MultiSCED/, or https://manolov.shinyapps.io/SeveralAB/.
  - Construct a modified Brinley plot (via https://manolov.shinyapps.io/Brinley/) for comparing the effects for the four tiers and check whether these effects meet the requirement (for the current example) of at least 1-point average improvement and at least an average of 0 in the Goal Attainment Scale after the intervention.
  - Assess the consistency in variability using the visual aid from https://tamalkd.shinyapps.io/scda. There are different ways of representing within-phase variability in this website; here we use the range.
  - Compute a success rate on the basis of an a priori criterion regarding what represents a positive result and the focal data feature. Focusing on overlap and NAP, it is possible to follow three criteria for a positive effect. The most lenient one is a NAP value of more than 50%, suggesting there is at least some difference between the conditions. An intermediate option is the NAP value of a moderate effect: 65% as per Parker and Vannest (2009). The most stringent option is to consider the maximum value^5^ (100%) as the threshold, since it is the value that represents complete separation (i.e., all intervention phase measurements show an improvement to all baseline phase measurements). Here we chose the threshold for a moderate effect (i.e., a value of 65%).
  - Complement with quantifications of variability across A–B comparisons, such as variance estimates from a two-level model (http://34.251.13.245/MultiSCED/) or the intraclass correlation from the between-case standardized mean difference (https://jepusto.shinyapps.io/scdhlm/). It should be noted that while the variance (or standard deviation) of the effect, as quantified via a multilevel model, refers specifically to the variability of the intervention effect, the intraclass correlation from the between-case standardized mean difference does not. Instead, the latter provides a quantification of the data variability that is across participants, and this data variability can also be in terms of the level of the behavior in experimentally similar phases. In that sense, the intraclass correlation mixes the two kinds of consistency outlined by Kratochwill and colleagues (2010, 2013): consistency of data in similar phases and consistency of effects. Proposals regarding the former kind of consistency are made by Tanious et al. (2020, 2021).

### Required data files

**Fig. 2:**
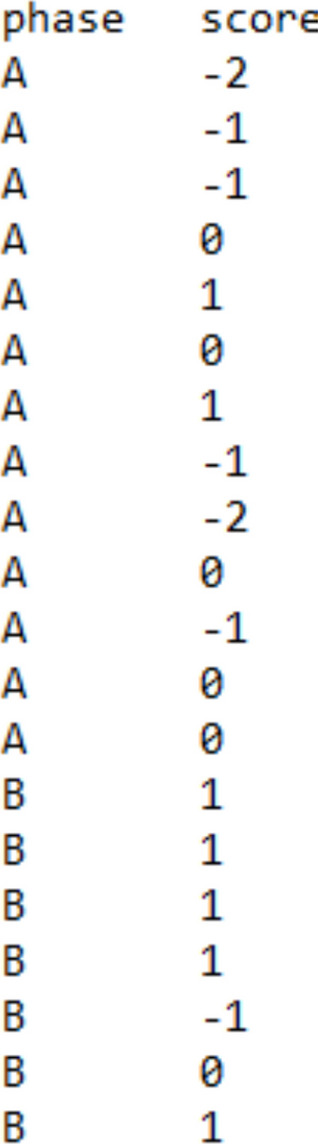
Data structure for using the websites for evaluating a basic effect (i.e., an A–B comparison)

For the basic effect (i.e., each A–B comparison), the websites require a simple .txt file with two columns: one that includes the measurements and one that includes the condition, marked with either A (baseline) or B (intervention). For https://tamalkd.shinyapps.io/scda/, it is better that the two columns contain no headers. In contrast, for the remaining websites (https://manolov.shinyapps.io/Overlap/; https://manolov.shinyapps.io/TrendMASE/; https://manolov.shinyapps.io/Regression/ and https://manolov.shinyapps.io/TrendMAD/), it is important to use the headers “score” (for the measurements of the target behavior) and “phase” (for the A and B labels), both in small letters. See Fig. 2 as an example.

**Fig. 3:**
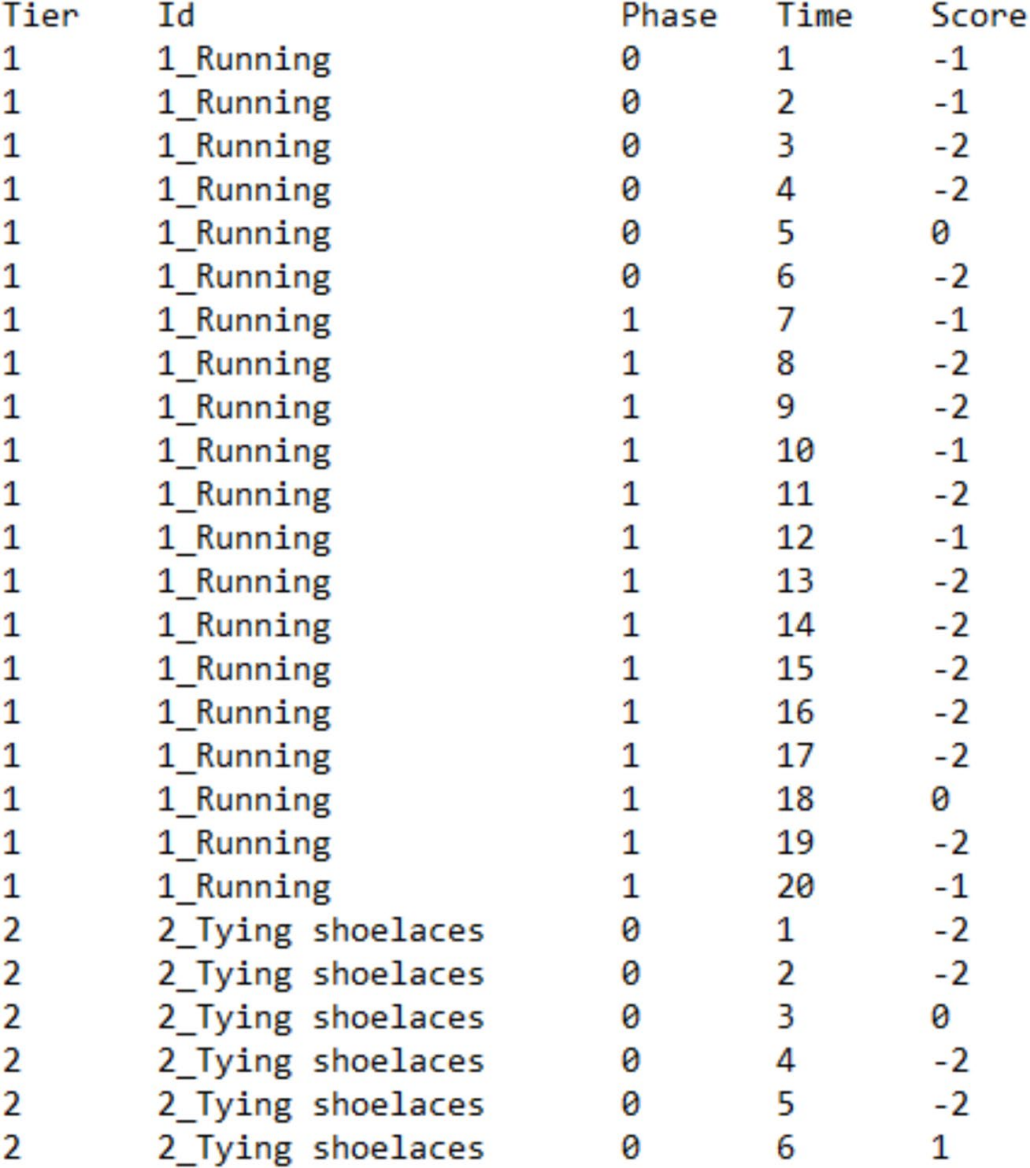
Data structure for using the websites for evaluating consistency using a multilevel model (i.e., several A–B comparisons)

For the assessment of consistency, the data files need to include more columns. Specifically, for https://manolov.shinyapps.io/Overlap/ (which allows superimposable one-level regression lines specific for each basic effect and two-level lines representing the average for all basic effects), the data file should be created as shown in Fig. 3. The first column (Tier) includes an integer identifying each of the A–B comparisons. The second column (Id) provides a more informative label on this comparison. The third column (Phase) marks the baseline condition with a 0 and the intervention with a 1. The fourth column (Time) marks the session number. The fifth column (Score) includes the measurement of the dependent variable.

**Fig. 4:**
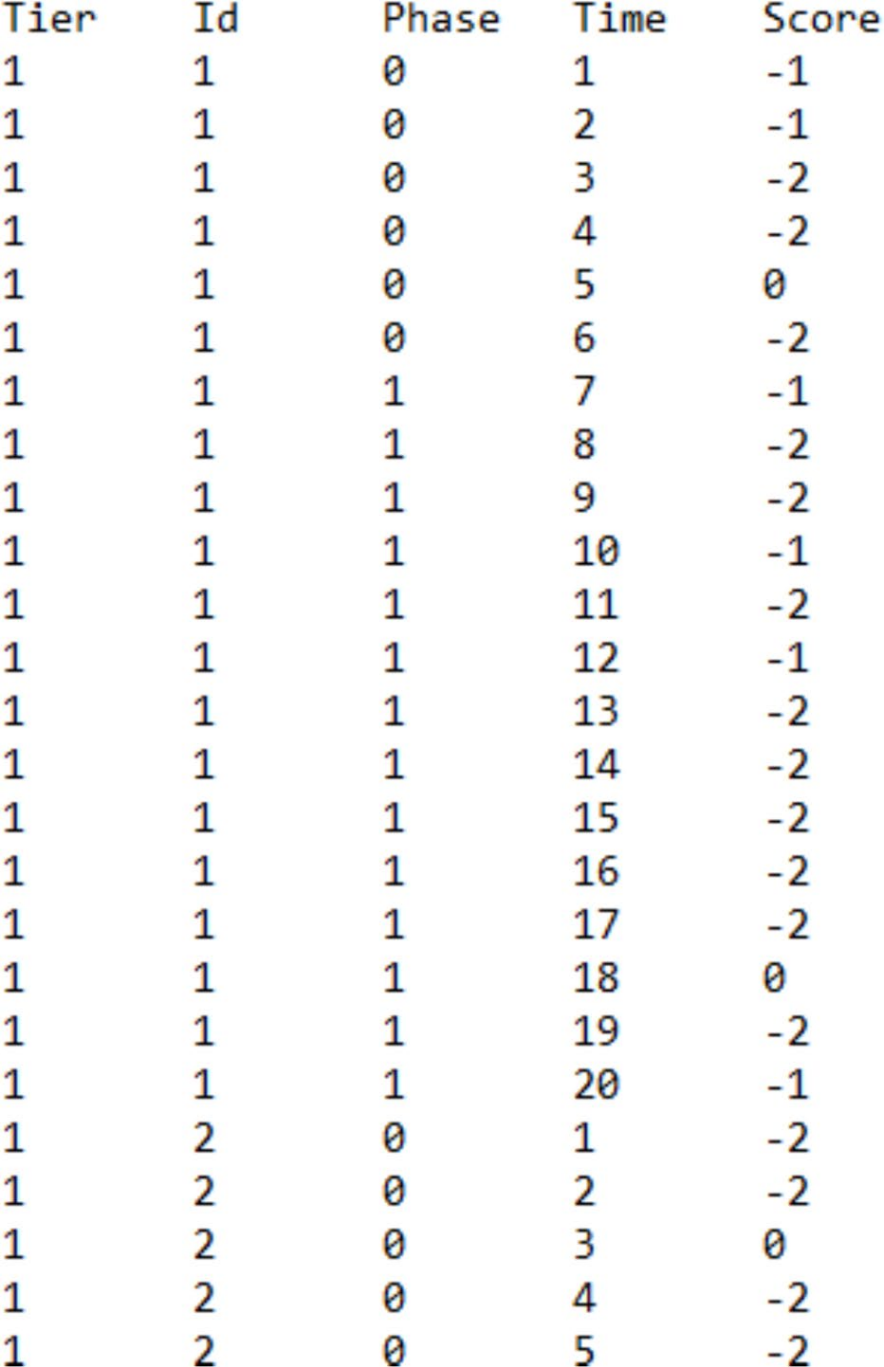
Data structure for using the website implementing the modified Brinley plot for evaluating consistency (i.e., several A–B comparisons)

For using https://manolov.shinyapps.io/Brinley/, the structure is practically identical (see Fig. 4). Specifically, the columns with the headers Phase, Time, and Score are defined as previously. The definitions of the columns Tier and Id are different, because this website is designed to handle several comparisons (i.e., different integers in the Id column) for several participants (i.e., different integers in the Tier column). Regarding the example data from the Lebrault et al. (2024) study, we could have depicted the four behaviors studied for the four participants. Given that the current focus is on one of the participants (Ian), the column Tier will only contain the integer 1, whereas the column Id will contain integers from 1 to 4 for the four behaviors.

**Fig. 5:**
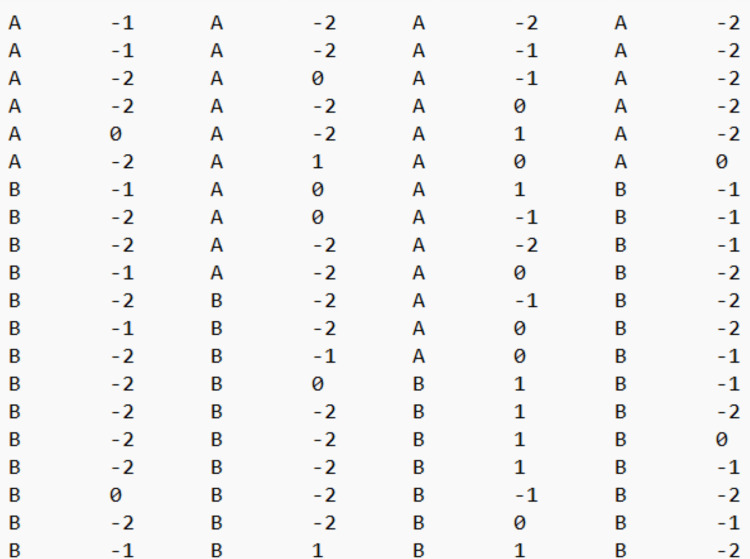
Data structure for using https://tamalkd.sninyapps.io/scda for visual analysis superimposing visual aids (or for randomization tests and computing quantifications of effect)

For using https://tamalkd.shinyapps.io/scda, for representing all of Ian’s data (i.e., the four goals), the data structure is as represented in Fig. 5: each column with a phase label is followed by a column with the measurements of the dependent variable, without headers.

For quantifications using the between-case standardized mean difference (Shadish et al., 2014) or multilevel model (Ferron et al., 2009, 2010), the data structure represented in Fig. 3 or 4 can be used for the former (https://jepusto.shinyapps.io/scdhlm/) and the structure from Fig. 4 for the latter (http://34.251.13.245/MultiSCED/). These analytical options offer not only average effect sizes but also quantifications of variability useful for assessing consistency (potentially useful for the assessment of consistency as part of the evaluation of functional relations). All data files included in the current tutorial can be accessed via https://osf.io/8ngwc/overview?view_only=4f3ac8bfc86544d49abd25a351ac2623.

## Results: Use of the software

### Basic effect: Two-phase comparison

**Fig. 6:**
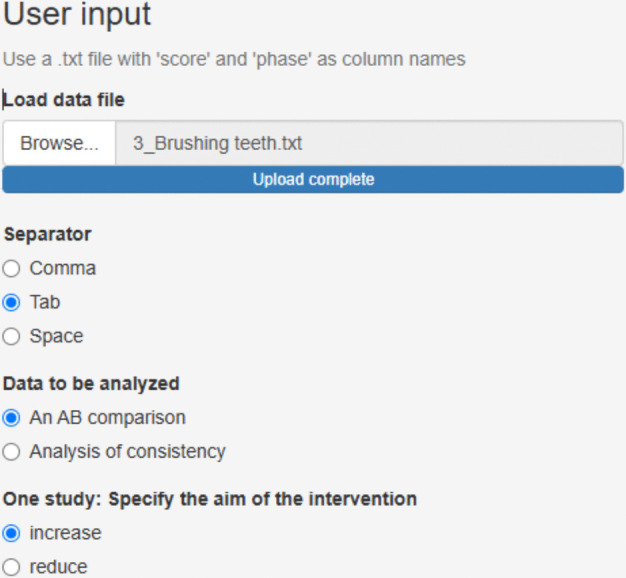
Menu from https://manolov.shinyapps.io/Overlap/*,* for locating the data file and defining the aim of the A–B comparison

**Fig. 7:**
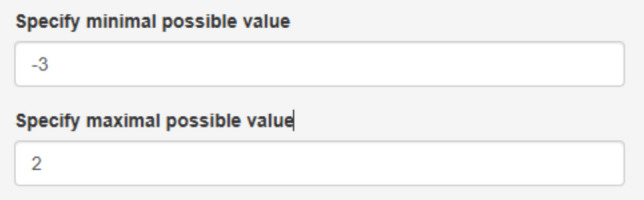
Menu from https://manolov.shinyapps.io/Overlap/, for defining the *y*-axis limits of the graphical representations, according to the way in which the dependent variable is measured

We focus on the third treated behavior for Ian, brushing teeth. After accessing https://manolov.shinyapps.io/Overlap/, the user has to browse the data file and specify that, in this case, the aim is to reduce the target behavior (Fig. 6). For optimal graphical representations, the lower and upper limits of the measure of the target behavior are specified (Fig. 7).

#### Level and trend: Overall, immediacy, and projection considering data variability

**Fig. 8:**
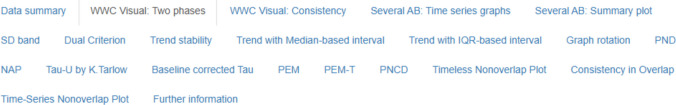
Tabs from https://manolov.shinyapps.io/Overlap/, for choosing different graphical representations, visual aids, and quantifications

On this website, the different graphical representations, visual aids, and quantifications are accessed via tabs. An overall representation of five data features (level, trend, variability, immediacy, and overlap) is obtained by clicking on the “WWC Visual: Two phases” tab (Fig. 8).

**Fig. 9:**
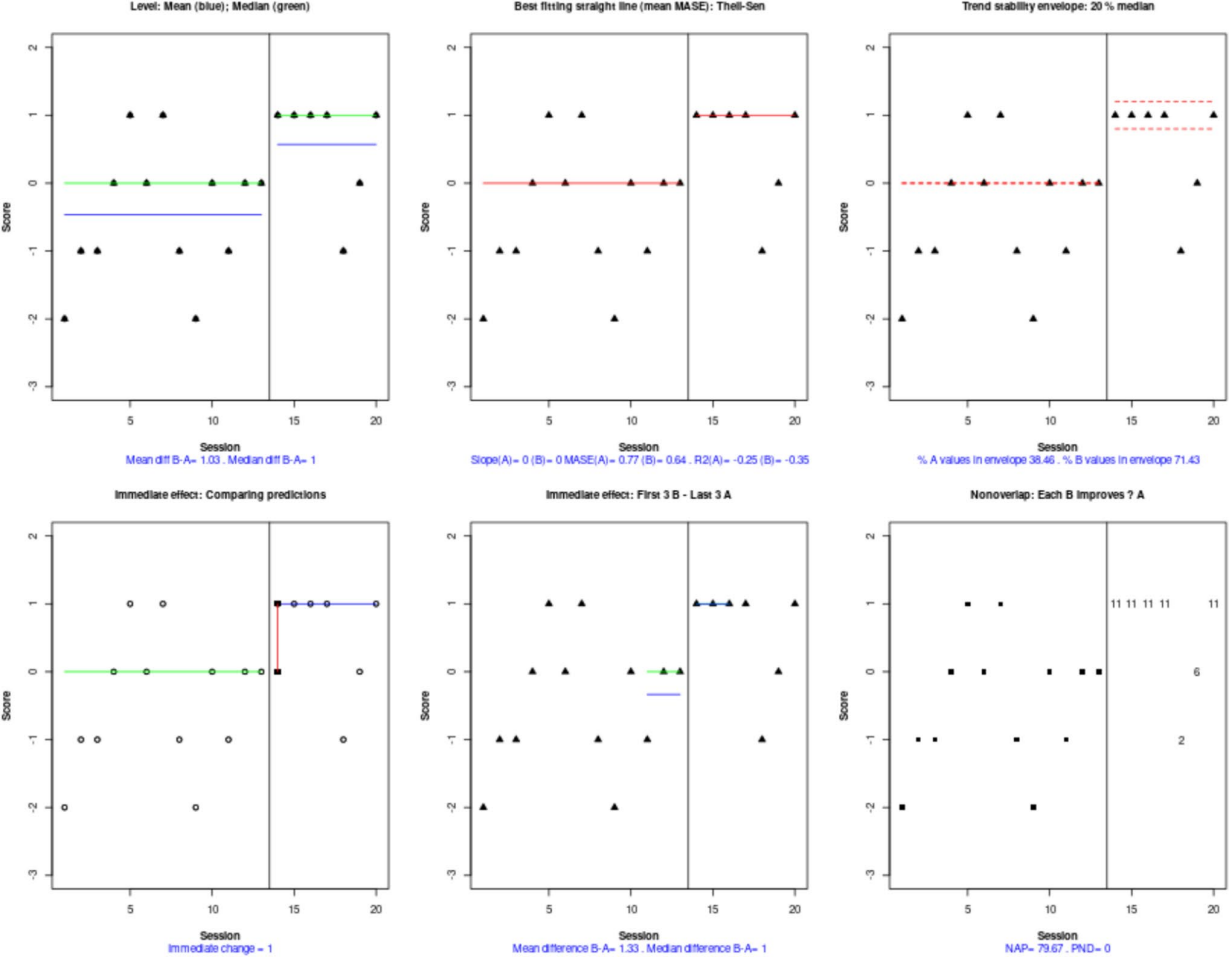
Graphical representation of five data features (level, trend, variability, immediacy, and overlap) from https://manolov.shinyapps.io/Overlap/

**Fig. 10:**
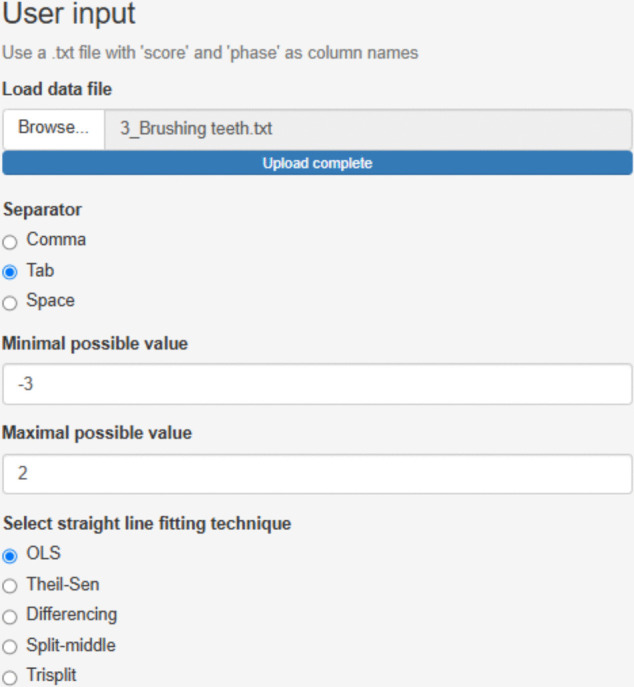
Menu of https://manolov.shinyapps.io/TrendMASE/

**Fig. 11:**
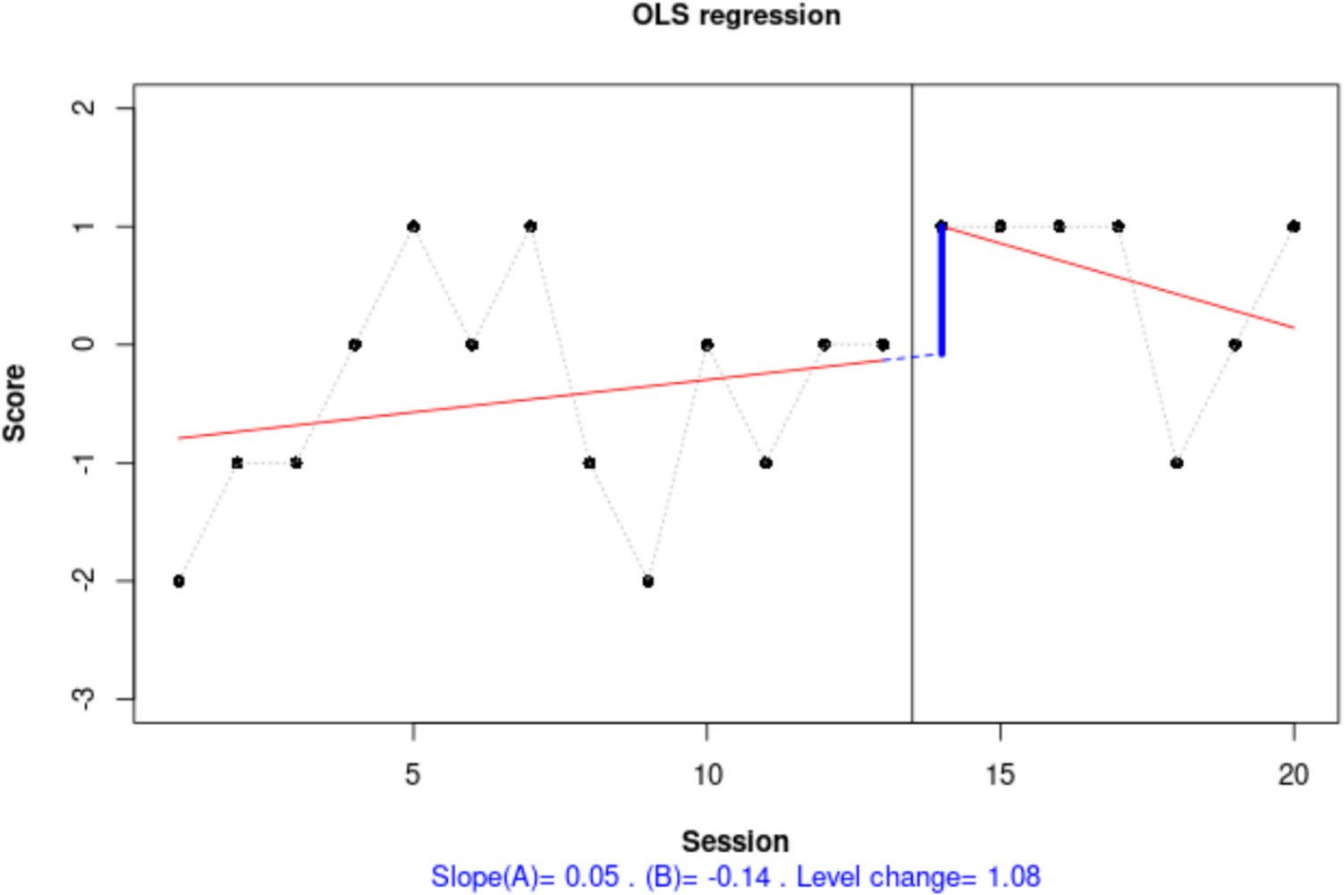
Graphical representation of within-phase linear trend estimated via ordinary least squares, obtained from https://manolov.shinyapps.io/TrendMASE/

This overall representation (Fig. 9) suggests substantial within-phase variability; as shown in the top right panel, less than 80% of data are within each stability envelope (Lane & Gast, 2014). This precludes us from drawing conclusions related to level^6^ and trend. In any case, if there were interest in a deeper assessment of trend, it should be noted that the website selected the Theil–Sen method for fitting a trend line for these data, following the criterion of the mean absolute scaled error (Hyndman & Koehler, 2006; Manolov et al., 2019). According to the trend lines fitted, there is no change in the measurements with time in either of the phases. However, it is possible to compare different trend lines via another website (https://manolov.shinyapps.io/TrendMASE) using the same data file (see Fig. 10 for specifying the *y*-axis of the plot and selecting a trend line fitting technique). For instance, using ordinary least squares estimation (Fig. 11), the trend lines of the two phases are not flat. Instead, the baseline trend is increasing (improving), whereas the intervention phase trend is decreasing (deteriorating), suggesting an overall deterioration according to this data feature and disregarding the immediate increase in level.

**Fig. 12:**
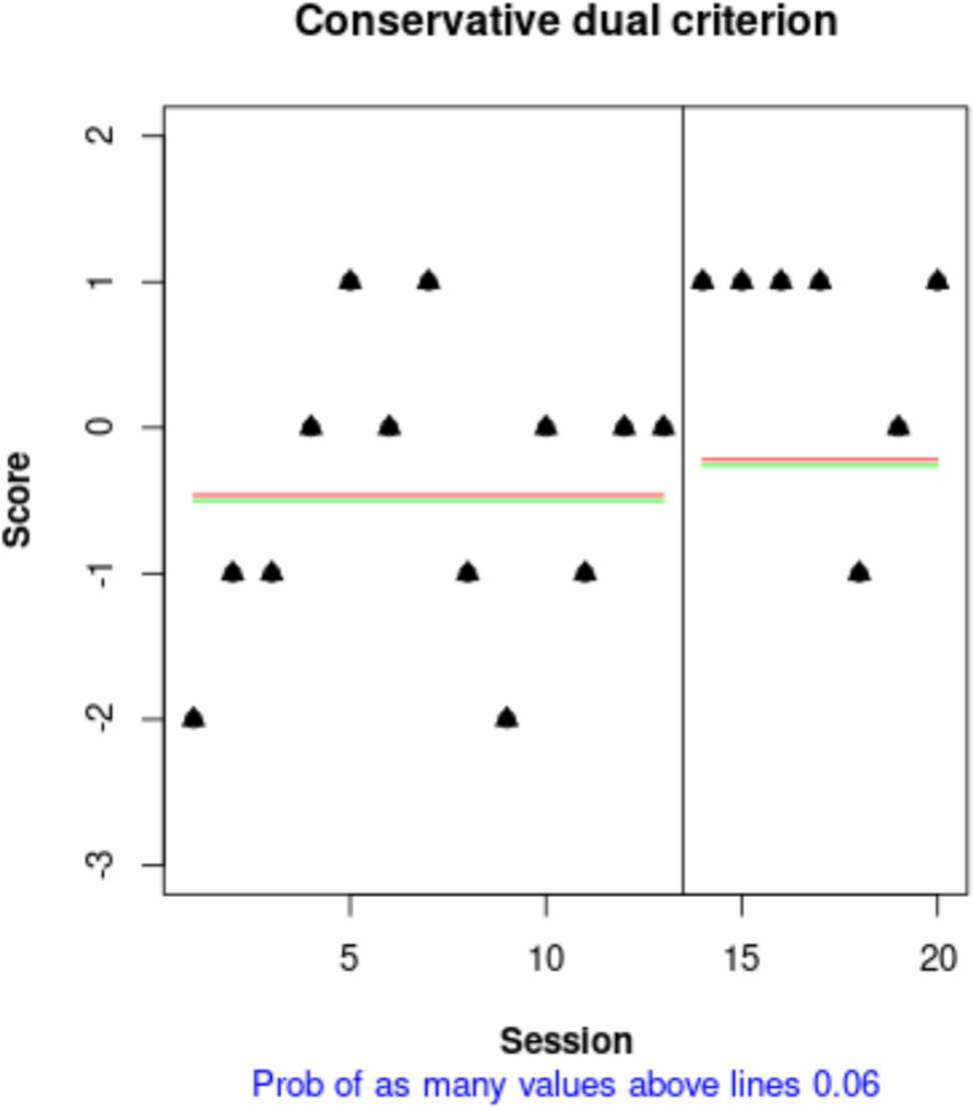
Graphical representation of the conservative dual criteria from https://manolov.shinyapps.io/Overlap/

If the aim were to compare the projected baseline with the actual intervention phase measurements, the conservative dual criteria combining the mean level and the split-middle trend (Fisher et al., 2003) could be used via the “Dual Criterion” tab of https://manolov.shinyapps.io/Overlap/. The result is depicted in Fig. 12 and suggests that the probability of six out of seven intervention phase measurements being above both lines only by change is .06, which is almost at the .05 threshold suggesting the presence of an intervention effect.

**Fig. 13:**
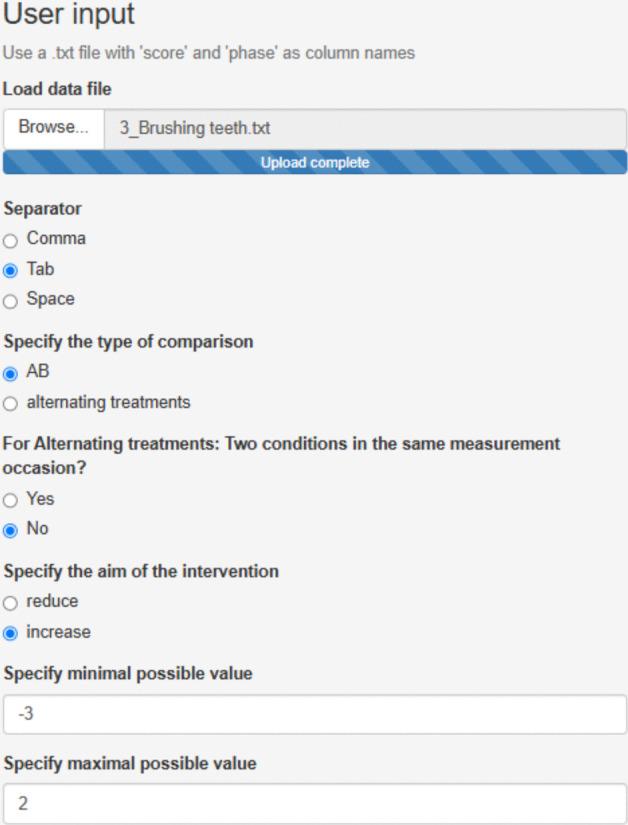
Menu of https://manolov.shinyapps.io/TrendMAD/

**Fig. 14:**
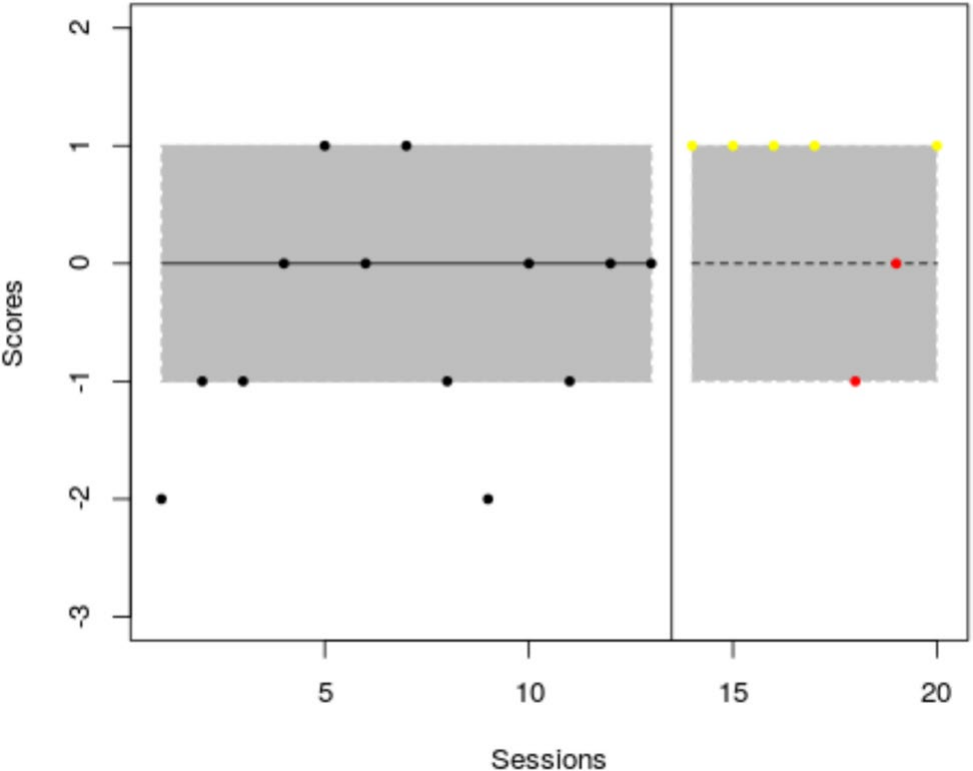
Graphical representation of projecting baseline trend with a variability band, obtained via https://manolov.shinyapps.io/TrendMAD/

However, it is also possible to take the variability into account when projecting baseline trend (see Manolov & Vannest, 2023) via https://manolov.shinyapps.io/TrendMAD. The menu is represented in Fig. 13 and the output in Fig. 14. This option indicates that all intervention phase measurements fall within the expected variability band, indicating no change.

#### Overlap

**Fig. 15:**
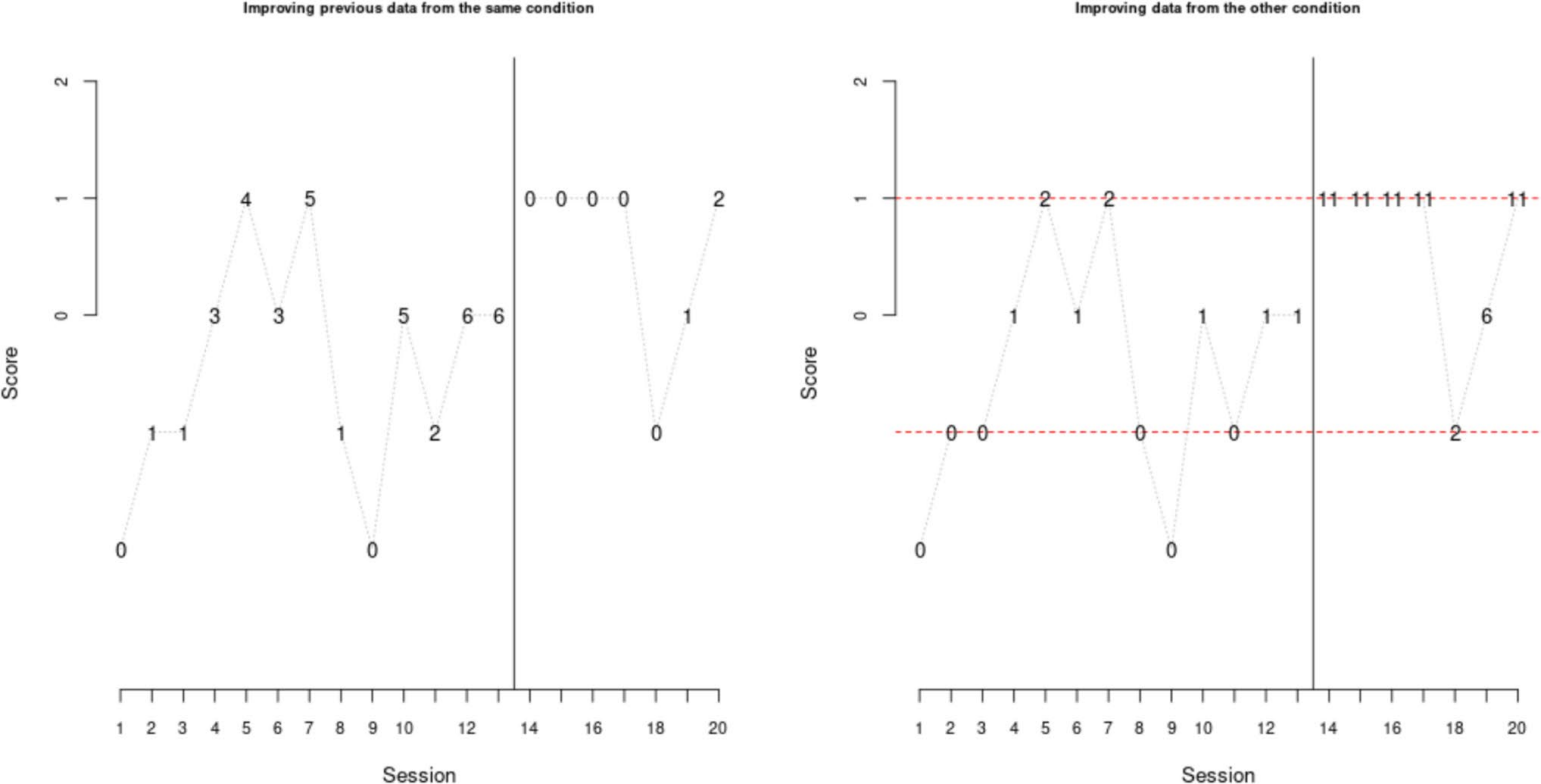
Result from https://manolov.shinyapps.io/Overlap/, for representing overlap

For the current example, we had established that the main interest lies in overlap due to the expected (and subsequently, actually observed) data variability. It can be considered that when data are highly variable, they may not be well represented by a straight line (such as a mean or a trend line), as would be indicated by a large error (bouncing) around such a line. In contrast, NAP compares the data points directly without reducing them to a summary line. In that sense, the preceding section is included only for illustrative purposes, and should not be the focus of the analysis. The https://manolov.shinyapps.io/Overlap/ website yields a NAP value of 80%, representing a moderate effect (between 65% and 92%, as per Parker and Vannest, 2009). Figure 15 provides further details on this quantification. Looking at the left plot, we see that there is no clear evidence for an improving monotonic baseline trend: although several measurements improve previous data points, this is not the case for all of them. Similarly, there is no clear monotonic trend in the intervention phase. Looking at the right plot, we see a large overlap area, but most of the intervention phase measurements represent improvements over (nonoverlaps with) most baseline data points. Thus, for this behavior, there seems to be an improvement in terms of overlap.

## Consistency: Visual inspection via a two-level model (level and trend)

**Fig. 16:**
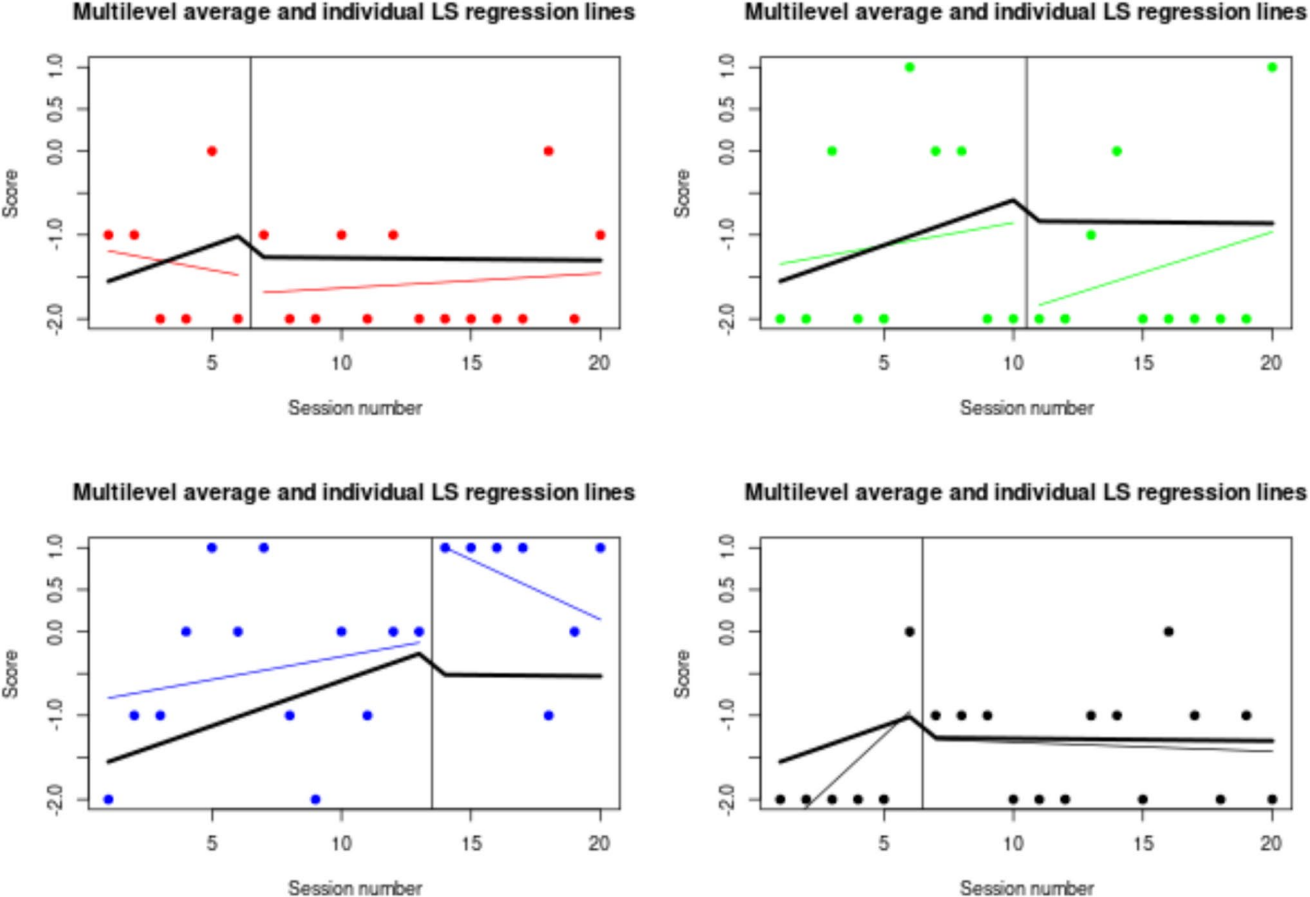
Result from https://manolov.shinyapps.io/Overlap/, for a visual inspection of consistency via a two-level model

For comparing the one-level regression trend lines (fitted for each behavior separately) and the average trend lines (obtained via a two-level model), the https://manolov.shinyapps.io/Overlap/ website can easily^7^ be used, as it offers an automatic depiction. Once the data (as represented in Fig. 3) are loaded, the output is obtained in the “WWC Visual: Consistency” tab (see Fig. 16). There is a considerable difference between the thick black lines (averages) and the colored lines representing each behavior: three of the baseline trends are increasing, whereas one is decreasing; two of the intervention phase trends are increasing, one is decreasing, and one is practically zero; the immediate effect is not consistently an increase or a decrease for all behaviors. Thus, at least visually, there is no evidence for consistency in either the trend lines or in the effect of the intervention.

## Consistency: Visual inspection via a modified Brinley plot (level)

**Fig. 17:**
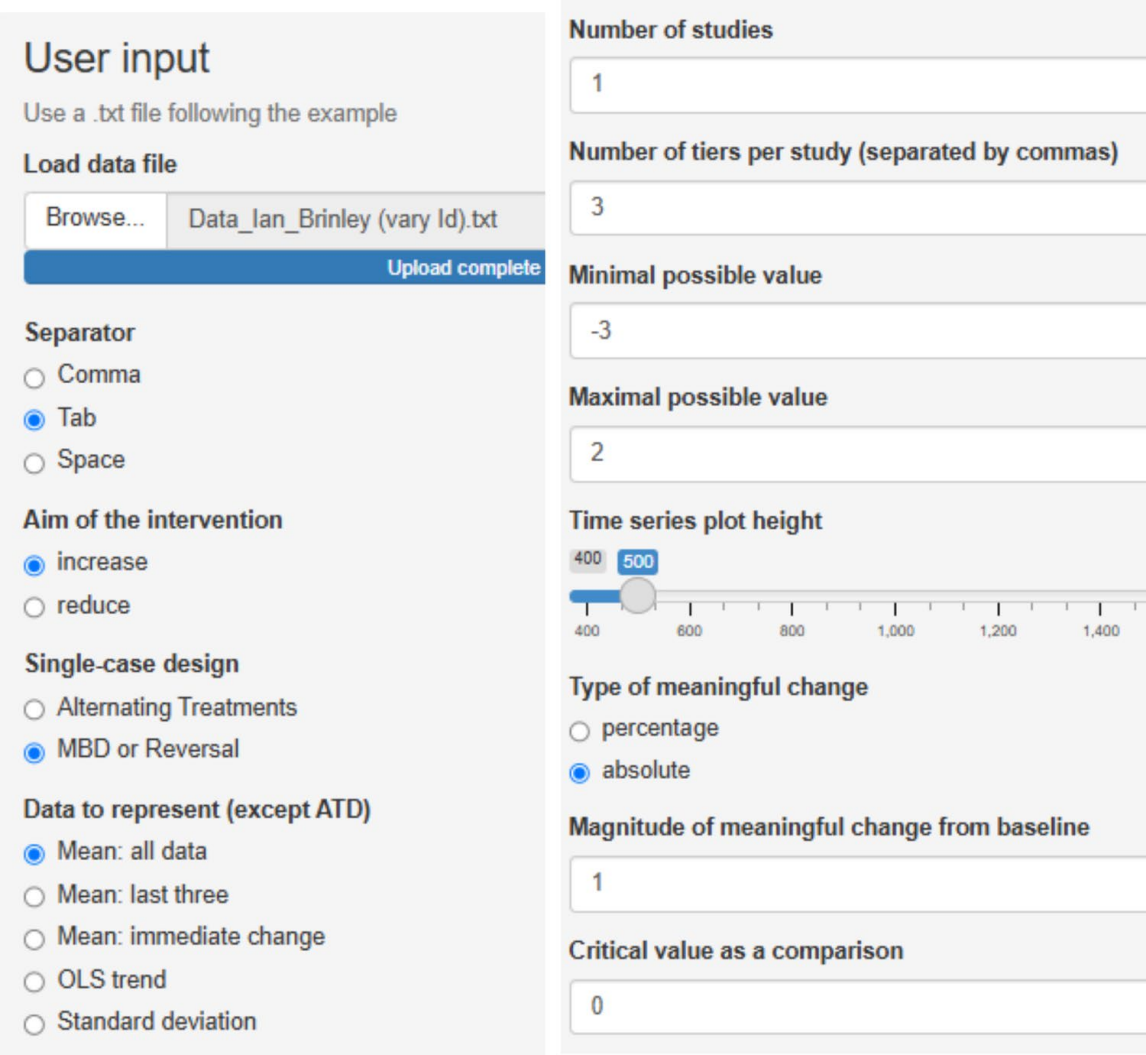
Menu from https://manolov.shinyapps.io/Brinley/

**Fig. 18:**
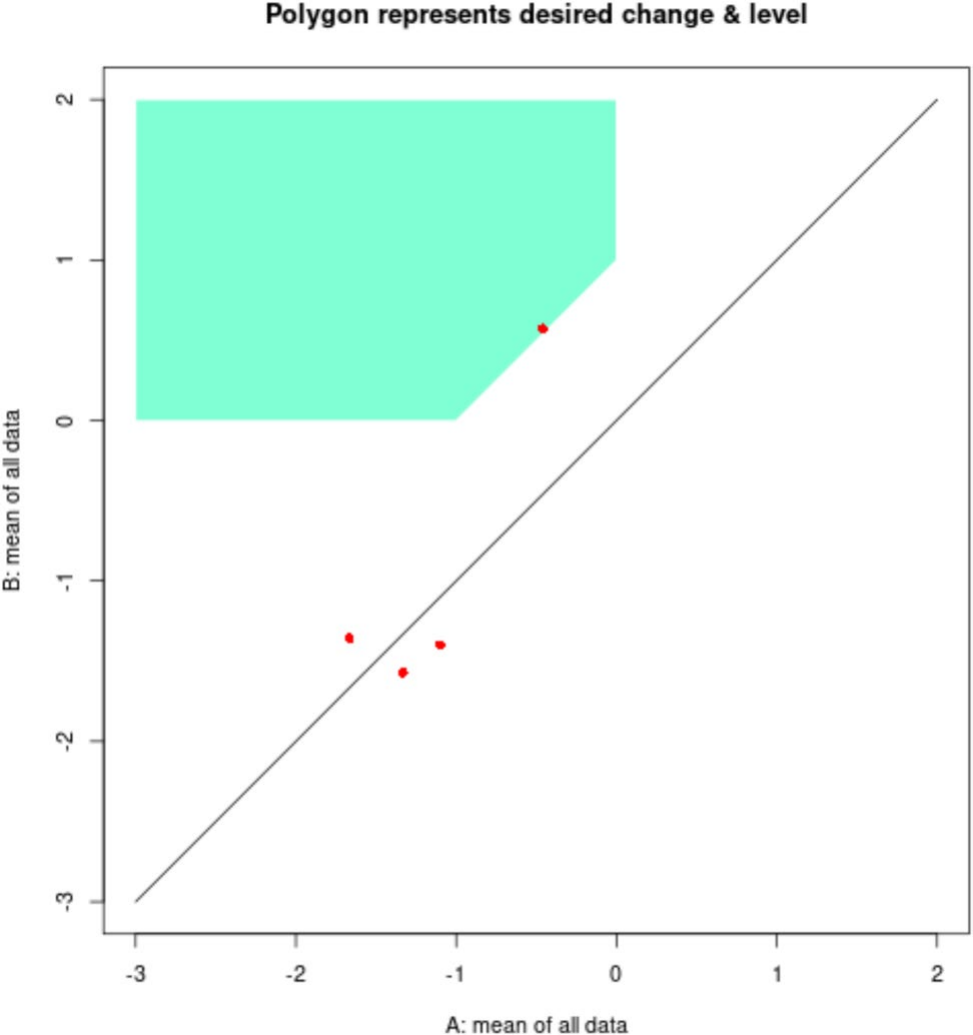
Modified Brinley plot with polygon for desired effect, from https://manolov.shinyapps.io/Brinley/

The data structured as shown in Fig. 4 are loaded in https://manolov.shinyapps.io/Brinley/. The menu is depicted in Fig. 17, including the definition of both axes according to the minimum and maximum values for the Goal Attainment Scale, the minimum required effect (1 point, in absolute terms) and the minimum required post-intervention mean (0, representing the desired goal). As per the “Main Brinley plot” tab (see Fig. 18), it can be seen that the dot is above the diagonal line for only two of the four tiers, representing an improvement (i.e., intervention phase mean higher than the baseline phase mean). Moreover, according to the colored polygon, only one of the dots (tiers) represents the desired amount of improvement. Clicking on the “Additional Brinley plots” tab, we can see that the improvements are the third behavior (brushing teeth) and the fourth one (i.e., the control behavior), with only the former presenting the desired amount of improvement. Thus, focusing on means, there is not enough evidence for a consistent effect.

## Consistency: Variability

**Fig. 19:**
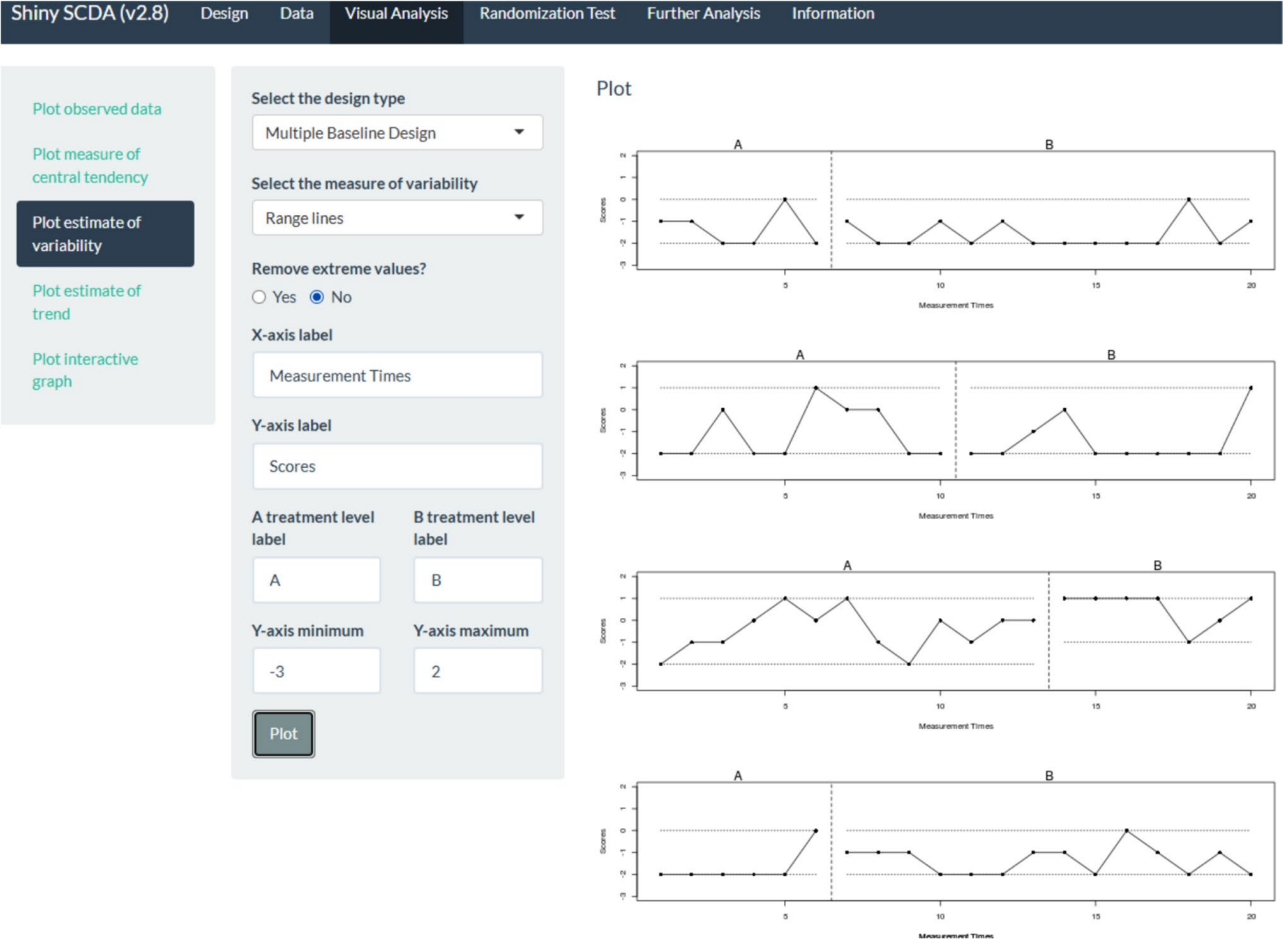
Menu from https://tamalkd.sninyapps.io/scda, alongside Ian’s data with range lines

If we focus on variability, using https://tamalkd.shinyapps.io/scda, we can see from Fig. 19 that the overall variability is larger (including almost all possible values between −3 and 2) for two of the behaviors than for the other two. In three of the comparisons, there is no difference in variability (operationally defined as the range) between the baseline and the intervention phase, and only for one behavior is there a reduction. Thus, what is consistent (although not desirable) is the presence of considerable data variability and an overall lack of reduction in variability after the intervention.

## Consistency: Success rate (overlap)

As stated previously, the current focus is on the NAP, as a quantification of overlap, with a requirement for a value of at least 65% (moderate effect, according to Parker & Vannest, 2009) for considering the effect positive. Considering that there are four opportunities to show an effect (i.e., four A–B comparisons) and that one of the behaviors is not treated, the expected (and desired) success ratio is 3:1. The use of https://manolov.shinyapps.io/Overlap/ for all four behaviors (with a data structure as depicted in Fig. 2) yields the following values: 42%, 44%, 80%, and 66%. Thus, only two of the basic effects can be considered a success (i.e., a 2:2 ratio), but the fourth behavior (NAP value of 66%) was control and was not expected to produce an effect. Therefore, there is not enough evidence for a consistent intervention effect on the basis of overlap as a data feature, just as was the case for means, described previously.

## Effect size calculation and quantifications of variability

Just for the sake of completeness, in addition to assessing the presence of a functional relation, we include the results of two possible quantifications: the between-case standardized mean difference (focusing on level via https://jepusto.shinyapps.io/scdhlm) and a two-level model incorporating trend and quantifying the change in slope and the immediate change in level (via https://34.251.13.245/MultiSCED/).

**Fig. 20:**
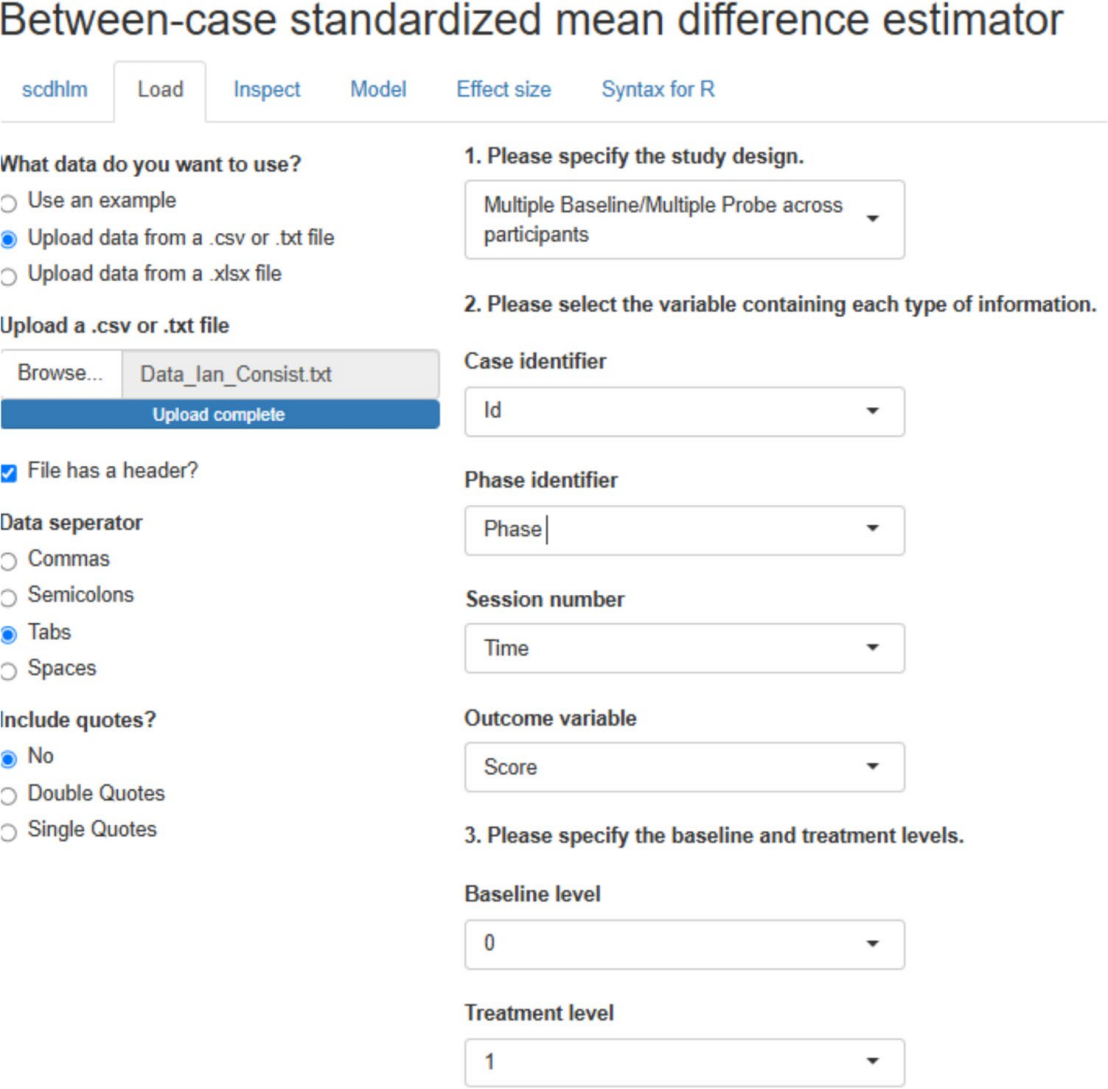
Menu of https://jepusto.shinyapps.io/scdhlm

Regarding the between-case standardized mean difference, the data are loaded in https://jepusto.shinyapps.io/scdhlm, and the columns are identified as specified in Fig. 20. The moment estimation^8^ yields an estimate of 0.1789 with a standard error of 0.2182, leading to a 95% confidence interval ranging between −0.2783 and 0.6362, suggesting that any average effect is likely to be irrelevant. Excluding the fourth (control) behavior, the effect estimate is even lower: 0.1234. Regarding variability across tiers (here, the different behaviors studied for Ian), the intraclass correlation is equal to 0.3205, suggesting considerable variability.

**Fig. 21:**
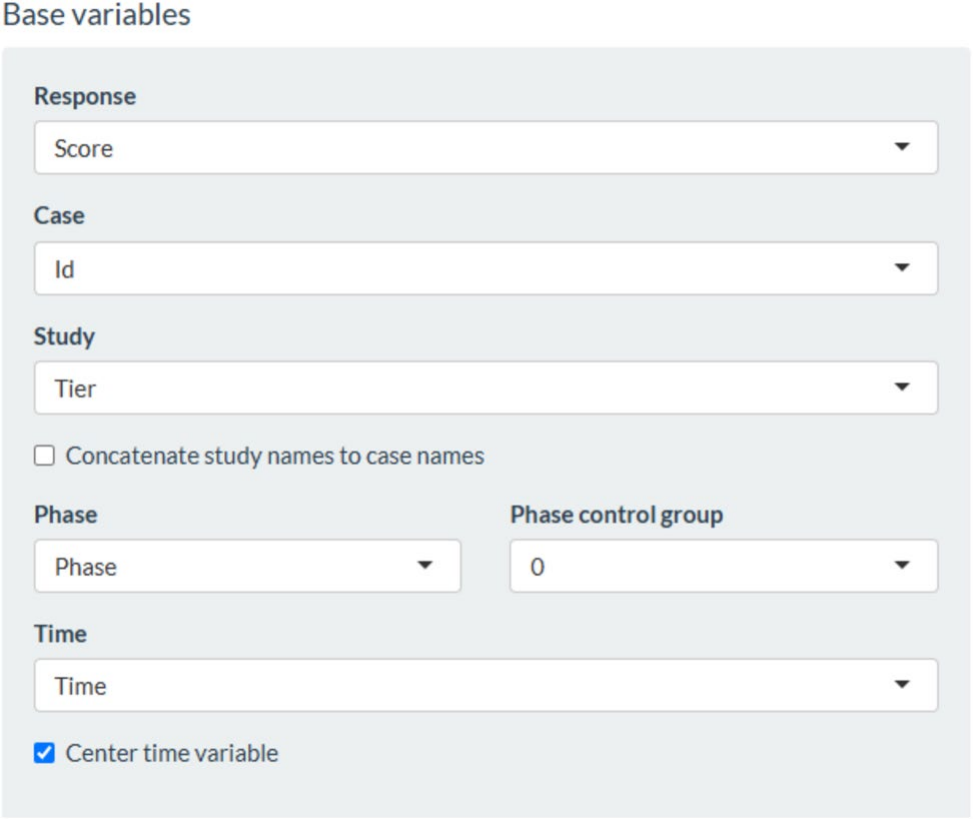
Menu of https://34.251.13.245/MultiSCED/ for identifying the variables

**Fig. 22:**
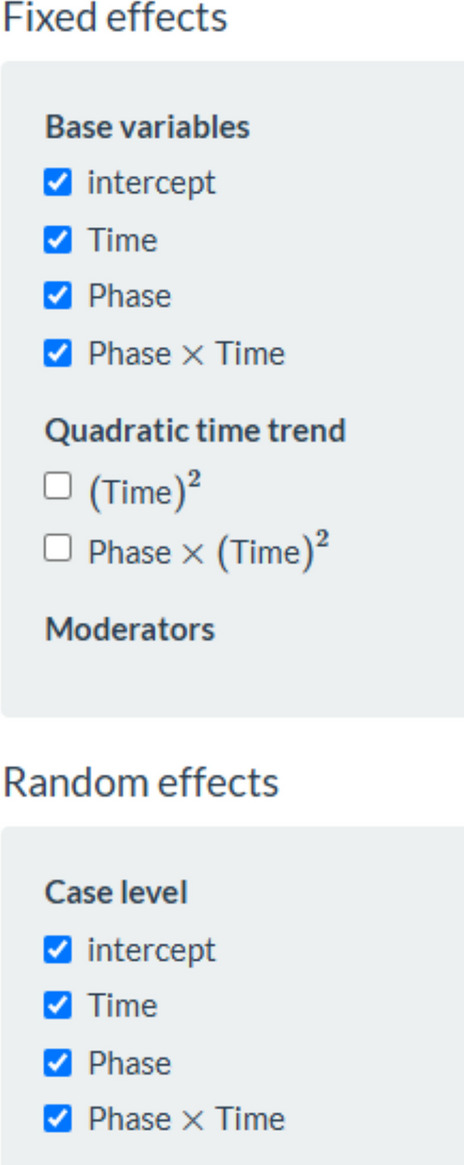
Menu of https://34.251.13.245/MultiSCED/ for specifying the model

Regarding a two-level model including separate estimates of baseline and intervention phase trend, the data required by the website https://34.251.13.245/MultiSCED/ are as shown in Fig. 4 (for the modified Brinley plot). The columns representing the different variables are identified as shown in Fig. 21. This specification includes centering the time variable so that, when modeling trend, the change in level is quantified for the first intervention phase measurement occasion. The model is specified as in Fig. 22, including separate estimates of trend for the baseline and the intervention phase and a quantification of change in level and immediate change in trend. The fixed effects represent the averages for the four behaviors, and the random effects quantify the variability around these averages. All these estimates are in the same measurement units as the dependent variable (the Goal Attainment Scale ranging from −3 to 2) rather than being standardized.

**Fig. 23:**
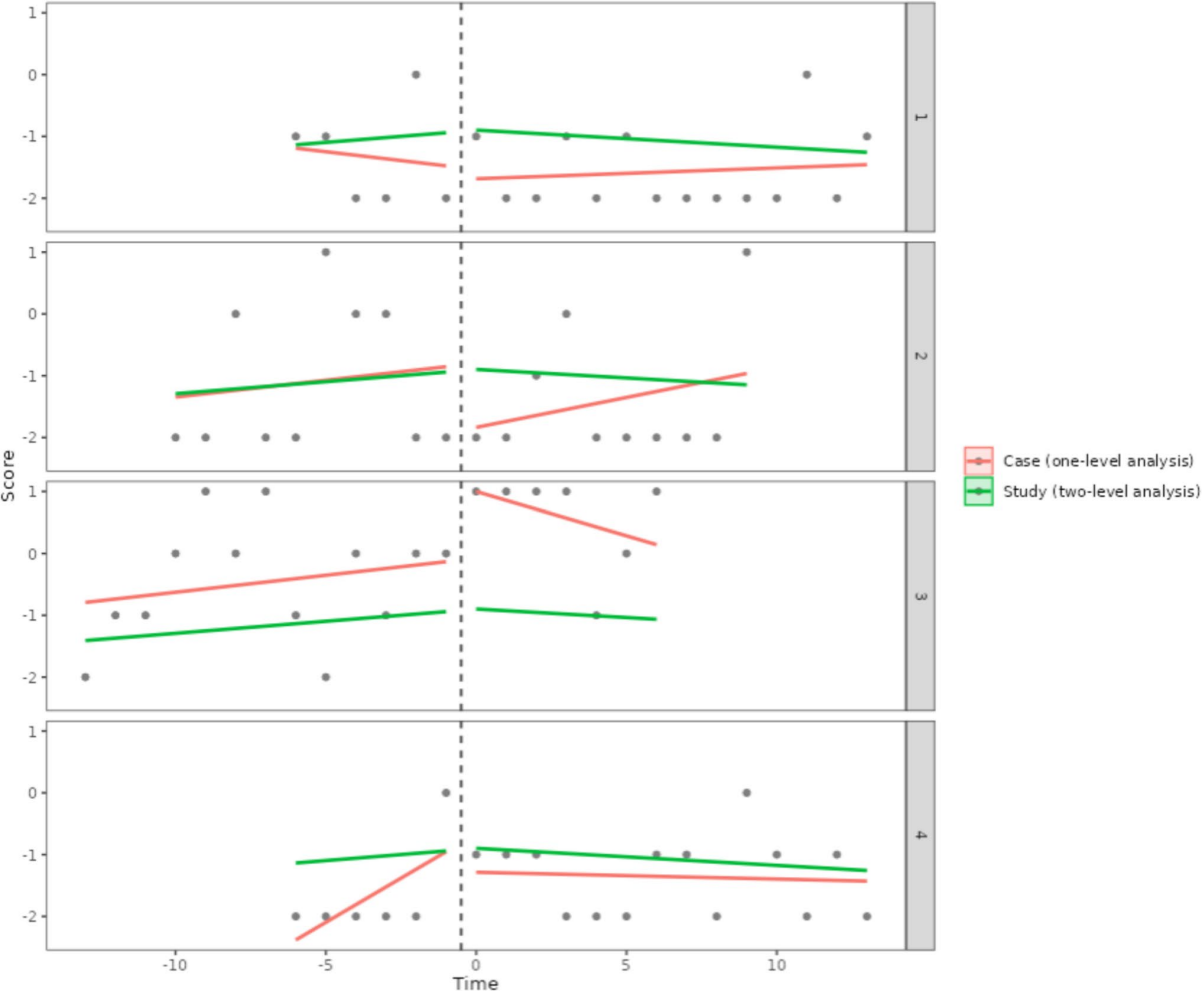
Graphical results from https://34.251.13.245/MultiSCED/

The results suggest practically zero immediate effect on average and an average decrease in trend after the intervention of 0.06 (after an average positive baseline trend of 0.03). The variability estimates, expressed as standard deviations, are as follows: for baseline trend, 0.008 (suggesting similar baseline trends), for the immediate effect of the intervention, 0.697 (suggesting great variability across behaviors), and for the difference in trend, 0.090 (also suggesting considerable variability, when compared to the average value). A graphical representation of the ordinary least squares regression lines fitted separately for each behavior (in red) and the two-level averages (in green) is available in Fig. 23. Thus, the conclusion of this analysis is equivalent to the previous one: no evidence for a consistent effect, given (mainly) the variability observed across goals and (additionally) the small estimates of effect. A necessary subsequent step would be to study why the intervention was effective for some behaviors but not for others.

## Discussion

### Success rate

Identifying evidence-based practices has been strongly related to performing quantitative integrations of several studies on the same intervention and target behavior, even in the SCED context (Jenson et al., 2007). Such an integration can be performed by using multilevel models (Baek et al., 2023; Declercq et al., 2022; Moeyaert & Yang, 2021; Van den Noortgate & Onghena, 2003a, 2003b). Notwithstanding the importance of documenting the magnitude of effect, in the SCED context it is important to consider whether there is enough evidence that any difference observed (quantified) in the target behavior can be considered to be reliably (consistently) due to the intervention. This is where the assessment of functional relations becomes important.

As illustrated in the current text, such an assessment can be performed by visual analysis aided by several graphical aids and descriptive quantifications, and can also be summarized quantitatively via a success rate. It should be noted that counting favorable results resembles vote-counting as an integration procedure applicable for a meta-analysis of several studies (Bushman & Wang, 2009). Thus, just as with weighted means, there is a continuity with the common meta-analytical practice.

Regarding the need for further debate, the assessment of functional relations and the success rate both require us to define what a positive result is. We suggest that such a definition should not necessarily be completely automated by comparing a quantification to a universal (null, moderate, or maximum) cutoff point or benchmark. Instead, it is likely to require the researchers’ judgment (Branch, 2014; Hagopian, 2020; Imam, 2021) based on their knowledge of the participant and the issue studied.

### Transparent reporting

Part of transparent reporting for avoiding questionable research practices (Tincani et al., 2025) is to mention all data analytical decisions made, beyond following the relevant reporting guidelines (Tate et al., 2016). Following a data analytical plan as described here can be useful for that purpose. Moreover, in relation to replicability and the current tutorial, it is also important to report the software used for visual representation, visual aids, and all quantifications obtained.

Similarly, reporting null or negative results is important, as long as they stem from methodologically sound studies, for at least two reasons (Kratochwill et al., 2018; Tincani & Travers, 2018). One the one hand, it is important to avoid publication bias and inflating effect estimates. On the other hand, it is relevant to identify what works (and does not work) for whom, and for that purpose, moderator analysis can be especially useful (Moeyaert & Yang, 2021).

### Limitations and additional resources

The current text focuses on the classical features which are usually the object of visual analysis as performed by a human researcher, as described in several sources: Kratochwill et al. (2010), Lane and Gast (2014), Maggin et al. (2018), Ledford et al. (2019). In contrast, it does not include another option for reaching a conclusion regarding the presence or absence of an intervention effect: the more or less autonomous use of computer-intensive methods or the use of artificial intelligence (Mensah & Chen, 2025). The reader interested in the latter can consult Lanovaz and Bailey (2024) for the use of artificial neural networks and Turgeon and Lanovaz (2020) for machine learning (in terms of websites for implementing this analytical option, see https://labrl.shinyapps.io/MachineLearningQABF/; https://labrl.shinyapps.io/MachineLearningABGraphs/; https://labrl.shinyapps.io/singlecaseanalysis/). Although here we echo previous claims on the importance of human judgment (Imam, 2021), applied researchers are encouraged to consult as much information as possible in order to decide for themselves how to approach the task of assessing the presence of a functional relation in SCED data.

Regarding the data features which are the object of visual analysis, it is important to mention that the current text did not deal with certain complexities related to trend. On the one hand, when projecting baseline trend, it is important to consider whether some extrapolations may be unreasonable or even impossible (Manolov et al., 2019; Parker, Vannest, Davis, & Sauber, 2011a, 2011b). On the other hand, even though the focus was linear trend, nonlinear data patterns can take place and be modeled (see Cheng et al., 2025; Hembry et al., 2015; Swan & Pustejovsky, 2018; Verboon & Peters, 2020).

Another simplification introduced in the text refers to consistency. While Kratochwill et al. (2010, 2013) referred to two kinds of consistency (of data in similar phases and of effects), in the current text the focus is on the latter, as it may be of greater interest for researchers. Nevertheless, proposals regarding the quantification of the consistency of data in experimentally similar phases can be consulted in Tanious et al. (2020, 2021; see the website https://manolov.shinyapps.io/CONDAP/ implementing these proposals).

The fact that the current tutorial includes multiple steps in the data analytical process and multiple websites can also be viewed as a limitation. The underlying rationale was to try to provide a comprehensive guide for a complex process. In any case, if simplifying (excessively?) is required, an applied researcher using SCEDs could focus on (a) performing visual analysis including all six data features (Kratochwill et al., 2010; Lane & Gast, 2014; Ledford et al., 2019) using https://manolov.shinyapps.io/Overlap/, or (b) multilevel modeling for obtaining an average quantification of effect alongside its variability (Ferron et al., 2009; Moeyaert, Ferron, et al., 2014a, 2014b) via http://34.251.13.245/MultiSCED/ (website described in Declercq et al., 2020) and the quantification of individual effects for each A–B comparison (Ferron et al., 2010) via https://manolov.shinyapps.io/SeveralAB/ (website discussed in Manolov & Moeyaert, 2025).

Finally, the six data features that are the object of visual analysis are mainly useful for multiple-baseline and reversal (ABAB) designs. In contrast, alternating-treatment designs in which there are only one or two consecutive measurements in the same condition (Barlow & Hayes, 1979) are assessed differently (see Diller et al., 2016; Lanovaz et al., 2019; Manolov & Onghena, 2018; some analytical options are implemented at https://tamalkd.shinyapps.io/scda and https://manolov.shinyapps.io/ATDesign). Similarly, the changing criterion design entails a comparison of the measurements to predefined desired levels (Hartmann & Hall, 1976). These designs are accompanied by specific  methodological recommendations (Klein et al., 2017) and data analytical approaches (Ferron et al., 2023; Manolov et al., 2020; Onghena et al., 2019). Some data plotting options for the changing criterion design are available at https://manolov.shinyapps.io/ChangingCriterion/). For both kinds of designs, the coding in multilevel modeling can be adapted (Shadish et al., 2013).

## Data Availability

The data used for the illustration can be downloaded from https://osf.io/8ngwc/overview?view_only=4f3ac8bfc86544d49abd25a351ac2623.

## Footnotes

1 In an alternating-treatment design, there are no phases, but there are still several A–B comparisons, and the pervasiveness of an effect can be quantified as the percentage of comparisons for which one condition is superior to the other (see Manolov & Onghena, 2018).

2 This is what is represented in a modified Brinley plot as presented by Blampied (2017), but it is also possible to represent a measure of trend or variability.

3 Specifically, data were retrieved from the online supplementary material found at: https://onlinelibrary.wiley.com/action/downloadSupplement?doi=10.1111%2Fdmcn.15759&file=dmcn15759-sup-0005-FigureS1.pdf

4 Here we refer to the classical and simpler proposals for assessing immediacy in the context of visual analysis. Nonetheless, other proposals have been made; see Natesan and Hedges (2017), Manolov and Onghena (2022).

5 In contrast to nonoverlap indices, other quantifications such as the one based on mean differences (e.g., Busk & Serlin, 1992; Carlin & Costello, 2018; Hedges et al., 2012, 2013; Pustejovsky, 2018) do not have such an upper bound, although certain interpretative benchmarks have been proposed specifically for the SCED context (Harrington & Velicer, 2015).

6 If we were forced to comment, we could say that there is an increase in level (in terms of both mean and, more appropriately for the ordinal data, median), as per the top left plot of Fig. 9. We could also say that this effect is immediate, according to the bottom left and center plots of Fig. 9.

7 In the “Effect size calculation and quantifications of variability” section, we will show how the http://34.251.13.245/MultiSCED/ website can also be used for that purpose, even though it requires some additional clicking.

8 The expression “moment estimation” refers to the way in which the quantification of effect is obtained when following the proposal by Hedges et al. (2013). It is also the label used in the website (https://jepusto.shinyapps.io/scdhlm). In contrast, the similar (but not equivalent) proposal for quantifying a standardized mean difference, as described by Pustejovsky et al. (2014), also available in the same website, relies on restricted maximum likelihood estimation.

## Code availability

Freely available web-based applications are illustrated. Workshop slides for applying different data analytical options to SCED data, including the websites reviewed in the text, can be downloaded from https://osf.io/t6ws6/.

## References

[CR1] Baek, E., Luo, W., & Kwon, H. L. (2023). Meta-analysis of single-case experimental design using multilevel modeling. *Behavior Modification,**47*(6), 1546–1573. 10.1177/01454455221144034 36647266 10.1177/01454455221144034

[CR2] Barlow, D. H., & Hayes, S. C. (1979). Alternating treatments design: One strategy for comparing the effects of two treatments in a single subject. *Journal of Applied Behavior Analysis,**12*(2), 199–210. 10.1901/jaba.1979.12-199 489478 10.1901/jaba.1979.12-199 PMC1311363

[CR3] Barton, E. E., Ledford, J. R., Lane, J. D., Decker, J., Germansky, S. E., Hemmeter, M. L., & Kaiser, A. (2016). The iterative use of single case research designs to advance the science of EI/ECSE. *Topics in Early Childhood Special Education,**36*(1), 4–14. 10.1177/0271121416630011

[CR4] Bishara, A. J., Peller, J., & Galuska, C. M. (2021). Misjudgment of interrupted time-series graphs due to serial dependence: Replication of Matyas and Greenwood (1990). *Judgment and Decision Making,**16*(3), 687–708. 10.1017/S1930297500007786

[CR5] Blampied, N. M. (2017). Analyzing therapeutic change using modified Brinley plots: History, construction, and interpretation. *Behavior Therapy,**48*(1), 115–127. 10.1016/j.beth.2016.09.002 28077215 10.1016/j.beth.2016.09.002

[CR6] Branch, M. (2014). Malignant side effects of null-hypothesis significance testing. *Theory & Psychology,**24*(2), 256–277. 10.1177/0959354314525282

[CR7] Bulté, I., & Onghena, P. (2013). The single-case data analysis package: Analysing single-case experiments with R software. *Journal of Modern Applied Statistical Methods,**12*, 450–478.

[CR8] Bushman, B. J., & Wang, M. C. (2009). Vote-counting procedures in meta-analysis. In H. Cooper, L. V. Hedges, & J. C. Valentine (Eds.), *The handbook of research synthesis and meta-analysis* (2nd Ed., pp. 207−220). Russell Sage.

[CR9] Busk, P. L., & Serlin, R. C. (1992). Meta-analysis for single-case research. In T. R. Kratochwill & J. R. Levin (Eds.), *Single-case research designs and analysis: New directions for psychology and education* (pp. 187–212). Lawrence Erlbaum.

[CR10] Carlin, M. T., & Costello, M. S. (2018). Development of a distance-based effect size metric for single-case research: Ratio of distances. *Behavior Therapy,**49*(6), 981–994. 10.1016/j.beth.2018.02.005 30316495 10.1016/j.beth.2018.02.005

[CR11] Carr, J. E. (2005). Recommendations for reporting multiple-baseline designs across participants. *Behavioral Interventions,**20*(3), 219–224. 10.1002/bin.191

[CR12] Cariveau, T., & Lewis, T. K. (2025). Omne trium perfectum/Everything that comes in threes is perfect. *Journal of Behavioral Education*. Advance online publication. 10.1007/s10864-025-09609-4

[CR13] Carter, M. (2013). Reconsidering overlap-based measures for quantitative synthesis of single-subject data: What they tell us and what they don’t. *Behavior Modification,**37*(3), 378–390. 10.1177/0145445513476609 23408384 10.1177/0145445513476609

[CR14] Center, B. A., Skiba, R. J., & Casey, A. (1985). A methodology for the quantitative synthesis of intra-subject design research. *The Journal of Special Education,**19*(4), 387–400. 10.1177/002246698501900404

[CR15] Cheng, K., Yi, Z., Moeyaert, M., Beretvas, S. N., Van den Noortgate, W., & Ferron, J. (2025). Synthesizing single-case experimental designs: Modeling complex data structures. *Journal of Behavioral Education*. Advance online publication. 10.1007/s10864-025-09602-x

[CR16] Cook, B. G., Buysse, V., Klingner, J., Landrum, T. J., McWilliam, R. A., Tankersley, M., & Test, D. W. (2015). CEC’s standards for classifying the evidence base of practices in special education. *Remedial and Special Education,**36*(4), 220–234. 10.1177/0741932514557271

[CR17] Cook, B. G., Johnson, A. H., Maggin, D. M., Therrien, W. J., Barton, E. E., Lloyd, J. W., . . . Travers, J. C. (2022). Open science and single-case design research. *Remedial and Special Education,**43*(5), 359–369. 10.1177/0741932521996452 10.1177/07419325211019100 PMC9426775 36052401

[CR18] Declercq, L., Cools, W., Beretvas, S. N., Moeyaert, M., Ferron, J. M., & Van den Noortgate, W. (2020). MultiSCED: A tool for (meta-)analyzing single-case experimental data with multilevel modeling. *Behavior Research Methods,**52*(1), 177–192. 10.3758/s13428-019-01216-2 30972557 10.3758/s13428-019-01216-2

[CR19] Declercq, L., Jamshidi, L., Fernández Castilla, B., Moeyaert, M., Beretvas, S. N., Ferron, J. M., & Van den Noortgate, W. (2022). Multilevel meta-analysis of individual participant data of single-case experimental designs: One-stage versus two-stage methods. *Multivariate Behavioral Research,**57*(2–3), 298–317. 10.1080/00273171.2020.1822148 32996335 10.1080/00273171.2020.1822148

[CR20] Diller, J. W., Barry, R. J., & Gelino, B. W. (2016). Visual analysis of data in a multielement design. *Journal of Applied Behavior Analysis,**49*(4), 980–985. 10.1002/jaba.325 27279559 10.1002/jaba.325

[CR21] Ferron, J. M., Bell, B. A., Hess, M. R., Rendina-Gobioff, G., & Hibbard, S. T. (2009). Making treatment effect inferences from multiple-baseline data: The utility of multilevel modeling approaches. *Behavior Research Methods,**41*(2), 372–384. 10.3758/BRM.41.2.372 19363177 10.3758/BRM.41.2.372

[CR22] Ferron, J. M., Farmer, J. L., & Owens, C. M. (2010). Estimating individual treatment effects from multiple-baseline data: A Monte Carlo study of multilevel-modeling approaches. *Behavior Research Methods,**42*(4), 930–943. 10.3758/BRM.42.4.930 21139160 10.3758/BRM.42.4.930

[CR23] Ferron, J. M., Goldstein, H., Olszewski, A., & Rohrer, L. (2020). Indexing effects in single-case experimental designs by estimating the percent of goal obtained. *Evidence-Based Communication Assessment and Intervention,**14*(1–2), 6–27. 10.1080/17489539.2020.1732024

[CR24] Ferron, J. M., Moeyaert, M., Van den Noortgate, W., & Beretvas, S. N. (2014). Estimating causal effects from multiple-baseline studies: Implications for design and analysis. *Psychological Methods,**19*(4), 493–510. 10.1037/a0037038 24933294 10.1037/a0037038

[CR25] Ferron, J., Rohrer, L. L., & Levin, J. R. (2023). Randomization procedures for changing criterion designs. *Behavior Modification,**47*(6), 1320–1344. 10.1177/0145445519847627 31081350 10.1177/0145445519847627

[CR26] Fisher, W. W., Kelley, M. E., & Lomas, J. E. (2003). Visual aids and structured criteria for improving visual inspection and interpretation of single-case designs. *Journal of Applied Behavior Analysis,**36*(3), 387–406. 10.1901/jaba.2003.36-387 14596583 10.1901/jaba.2003.36-387 PMC1284456

[CR27] Ganz, J. B., & Ayres, K. M. (2018). Methodological standards in single-case experimental design: Raising the bar. *Research in Developmental Disabilities,**79*(1), 3–9. 10.1016/j.ridd.2018.03.003 29655508 10.1016/j.ridd.2018.03.003

[CR28] Gilroy, S. P., Ledford, J. R., Elliott, T. C. C., Ayres, K. M., & McGill, F. (2025). Extending the Single Case Analysis and Review Framework (SCARF‐UI): A review and discussion. *Journal of Applied Behavior Analysis,**58*(4), 687–700. 10.1002/jaba.70033 41004415 10.1002/jaba.70033

[CR29] Grissom, R. J., & Kim, J. J. (2001). Review of assumptions and problems in the appropriate conceptualization of effect size. *Psychological Methods,**6*(2), 135–146. 10.1037/1082-989X.6.2.135 11411438 10.1037/1082-989x.6.2.135

[CR30] Hagopian, L. P. (2020). The consecutive controlled case series: Design, data‐analytics, and reporting methods supporting the study of generality. *Journal of Applied Behavior Analysis,**53*(2), 596–619. 10.1002/jaba.691 32125716 10.1002/jaba.691 PMC8805508

[CR31] Harrington, M., & Velicer, W. F. (2015). Comparing visual and statistical analysis in single-case studies using published studies. *Multivariate Behavioral Research,**50*(2), 162–183. 10.1080/00273171.2014.973989 26609876 10.1080/00273171.2014.973989 PMC4677800

[CR32] Hartmann, D. P., & Hall, R. V. (1976). The changing criterion design. *Journal of Applied Behavior Analysis,**9*(4), 527–532. 10.1901/jaba.1976.9-527 1002635 10.1901/jaba.1976.9-527 PMC1312042

[CR33] Hedges, L. V., Pustejovsky, J. E., & Shadish, W. R. (2012). A standardized mean difference effect size for single case designs. *Research Synthesis Methods,**3*(3), 224–239. 10.1002/jrsm.1052 26062165 10.1002/jrsm.1052

[CR34] Hedges, L. V., Pustejovsky, J. E., & Shadish, W. R. (2013). A standardized mean difference effect size for multiple baseline designs across individuals. *Research Synthesis Methods,**4*(4), 324–341. 10.1002/jrsm.1086 26053946 10.1002/jrsm.1086

[CR35] Hembry, I., Bunuan, R., Beretvas, S. N., Ferron, J. M., & Van den Noortgate, W. (2015). Estimation of a nonlinear intervention phase trajectory for multiple-baseline design data. *The Journal of Experimental Education,**83*(4), 514–546. 10.1080/00220973.2014.907231

[CR36] Heyvaert, M., & Onghena, P. (2014). Randomization tests for single-case experiments: State of the art, state of the science, and state of the application. *Journal of Contextual Behavioral Science,**3*(1), 51–64. 10.1016/j.jcbs.2013.10.002

[CR37] Horner, R. H., Carr, E. G., Halle, J., McGee, G., Odom, S., & Wolery, M. (2005). The use of single-subject research to identify evidence-based practice in special education. *Exceptional Children,**71*(2), 165–179. 10.1177/001440290507100203

[CR38] Horner, R. H., & Kratochwill, T. R. (2012). Synthesizing single-case research to identify evidence-based practices: Some brief reflections. *Journal of Behavioral Education,**21*(3), 266–272. 10.1007/s10864-012-9152-2

[CR39] Horner, R. H., & Odom, S. L. (2014). Constructing single-case research designs: Logic and options. In T. R. Kratochwill & J. R. Levin (Eds.), *Single-case intervention research: Methodological and statistical advances* (pp. 27–51). American Psychological Association. 10.1037/14376-002

[CR40] Hyndman, R. J., & Koehler, A. B. (2006). Another look at measures of forecast accuracy. *International Journal of Forecasting,**22*(4), 679–688. 10.1016/j.ijforecast.2006.03.001

[CR41] Imam, A. A. (2021). Historically recontextualizing Sidman’s Tactics: How behavior analysis avoided psychology’s methodological Ouroboros. *Journal of the Experimental Analysis of Behavior,**115*(1), 115–128. 10.1002/jeab.661 33336404 10.1002/jeab.661

[CR42] Johnson, A. H., & Cook, B. G. (2019). Preregistration in single-case design research. *Exceptional Children, 86*(1), 95–112. ://doi.org/10.1177/0014402919868529

[CR43] Jenson, W. R., Clark, E., Kircher, J. C., & Kristjansson, S. D. (2007). Statistical reform: Evidence-based practice, meta-analyses, and single subject designs. *Psychology in the Schools,**44*(5), 483–493. 10.1002/pits.20240

[CR44] Kazdin, A. E. (1977). Assessing the clinical or applied importance of behavior change through social validation. *Behavior Modification,**1*(4), 427–452. 10.1177/014544557714001

[CR45] Kinney, C. E., Dowdy, A., & Wolfe, K. (2025). A meta-visual-analysis of single-case experimental design research. *Behavior Modification,**49*(4), 343–376. 10.1177/01454455251320686 39972945 10.1177/01454455251320686

[CR46] Klein, L. A., Houlihan, D., Vincent, J. L., & Panahon, C. J. (2017). Best practices in utilizing the changing criterion design. *Behavior Analysis in Practice,**10*(1), 52–61. 10.1007/s40617-014-0036-x 28352507 10.1007/s40617-014-0036-x PMC5352619

[CR47] Kratochwill, T. R., Hitchcock, J., Horner, R. H., Levin, J. R., Odom, S. L., Rindskopf, D. M. & Shadish, W. R. (2010). Single-case designs technical documentation. Retrieved from What Works Clearinghouse website: https://ies.ed.gov/ncee/wwc/Docs/ReferenceResources/wwc_scd.pdf

[CR48] Kratochwill, T. R., Hitchcock, J. H., Horner, R. H., Levin, J. R., Odom, S. L., Rindskopf, D. M., & Shadish, W. R. (2013). Single-case intervention research design standards. *Remedial and Special Education,**34*(1), 26–38. 10.1177/0741932512452794

[CR49] Kratochwill, T. R., Horner, R. H., Levin, J. R., Machalicek, W., Ferron, J., & Johnson, A. (2021). Single-case design standards: An update and proposed upgrades. *Journal of School Psychology,**89*, 91–105. 10.1016/j.jsp.2021.10.006 34836578 10.1016/j.jsp.2021.10.006

[CR50] Kratochwill, T. R., Horner, R. H., Levin, J. R., Machalicek, W., Ferron, J., & Johnson, A. (2023). Single-case intervention research design standards: Additional proposed upgrades and future directions. *Journal of School Psychology,**97*, 192–216. 10.1016/j.jsp.2022.12.002 36914365 10.1016/j.jsp.2022.12.002

[CR51] Kratochwill, T. R., Levin, J. R., & Horner, R. H. (2018). Negative results: Conceptual and methodological dimensions in single-case intervention research. *Remedial and Special Education,**34*(1), 26–38. 10.1177/0741932512452794

[CR52] Lane, J. D., & Gast, D. L. (2014). Visual analysis in single case experimental design studies: Brief review and guidelines. *Neuropsychological Rehabilitation,**24*(3–4), 445–463. 10.1080/09602011.2013.815636 23883189 10.1080/09602011.2013.815636

[CR53] Lane, J. D., Ledford, J. R., & Gast, D. L. (2017). Single-case experimental design: Current standards and applications in occupational therapy. *American Journal of Occupational Therapy,**71*(2), 7102300010p1-7102300010p9. 10.5014/ajot.2017.022210 10.5014/ajot.2017.022210 28218597

[CR54] Lanovaz, M. J., & Bailey, J. D. (2024). Tutorial: Artificial neural networks to analyze single-case experimental designs. *Psychological Methods,**29*(1), 202–218. 10.1037/met0000487 35797162 10.1037/met0000487

[CR55] Lanovaz, M., Cardinal, P., & Francis, M. (2019). Using a visual structured criterion for the analysis of alternating-treatment designs. *Behavior Modification,**43*(1), 115–131. 10.1177/0145445517739278 29094610 10.1177/0145445517739278

[CR56] Laraway, S., Snycerski, S., Pradhan, S., & Huitema, B. E. (2019). An overview of scientific reproducibility: Consideration of relevant issues for behavior science/analysis. *Perspectives on Behavior Science,**42*(1), 33–57. 10.1007/s40614-019-00193-3 31976420 10.1007/s40614-019-00193-3 PMC6701706

[CR57] Lebrault, H., Martini, R., Manolov, R., Chavanne, C., Krasny-Pacini, A., & Chevignard, M. (2024). Cognitive orientation to daily occupational performance to improve occupational performance goals for children with executive function deficits after acquired brain injury. *Developmental Medicine and Child Neurology,**66*(4), 501–513. 10.1111/dmcn.15759 37792283 10.1111/dmcn.15759

[CR58] Ledford, J. R. (2018). No randomization? No problem: Experimental control and random assignment in single case research. *American Journal of Evaluation,**39*(1), 71–90. 10.1177/1098214017723110

[CR59] Ledford, J. R., Barton, E. E., Severini, K. E., & Zimmerman, K. N. (2019). A primer on single-case research designs: Contemporary use and analysis. *American Journal on Intellectual and Developmental Disabilities,**124*(1), 35–56. 10.1352/1944-7558-124.1.35 30715924 10.1352/1944-7558-124.1.35

[CR60] Ledford, J. R., Eyler, P. B., Windsor, S. A., & Chow, J. C. (2024). Single-case design effect-size distributions: Association with procedural parameters. *School Psychology,**39*(6), 589–600. 10.1037/spq0000636 38780589 10.1037/spq0000636

[CR61] Levin, J. R., Evmenova, A. S., Gafurov, B. S. (2014). The single-case data-analysis ExPRT (Excel Package of Randomization Tests). In T. R. Kratochwill & J. R. Levin (Eds.), *Single-case intervention research: Methodological and statistical advances* (pp. 185–219). American Psychological Association. 10.1037/14376-007

[CR62] Levin, J. R., Ferron, J. M., & Gafurov, B. S. (2017). Additional comparisons of randomization-test procedures for single-case multiple-baseline designs: Alternative effect types. *Journal of School Psychology,**63*, 13–34. 10.1016/j.jsp.2017.02.003 28633936 10.1016/j.jsp.2017.02.003

[CR63] Levin, J. R., Ferron, J. M., & Gafurov, B. S. (2021). Investigation of single-case multiple-baseline randomization tests of trend and variability. *Educational Psychology Review,**33*(2), 713–737. 10.1007/s10648-020-09549-7

[CR64] Levin, J. R., & Kratochwill, T. R. (2021). Randomized single-case intervention designs and analyses for health sciences researchers: A versatile clinical trials companion. *Therapeutic Innovation & Regulatory Science,**55*(4), 755–764. 10.1007/s43441-021-00274-z 33797058 10.1007/s43441-021-00274-z PMC8015743

[CR65] Li, H., Baek, E., Luo, W., Du, W., & Lam, K. H. (2025). Using generalized linear mixed models in the analysis of count and rate data in single-case experimental designs: A step-by-step tutorial. *Evaluation & the Health Professions,**48*(1), 143–155. 10.1177/01632787241259500 39660841 10.1177/01632787241259500

[CR66] Maggin, D. M., Barton, E., Reichow, B., Lane, K., & Shogren, K. A. (2022). Commentary on the What Works Clearinghouse Standards and Procedures Handbook (v. 4.1) for the review of single-case research. *Remedial and Special Education,**43*(6), 421–433. 10.1177/07419325211051317

[CR67] Maggin, D. M., Briesch, A. M., & Chafouleas, S. M. (2013). An application of the What Works Clearinghouse standards for evaluating single-subject research: Synthesis of the self-management literature base. *Remedial and Special Education,**34*(1), 44–58. 10.1177/0741932511435176

[CR68] Maggin, D. M., Cook, B. G., & Cook, L. (2018). Using single‐case research designs to examine the effects of interventions in special education. *Learning Disabilities Research & Practice,**33*(4), 182–191. 10.1111/ldrp.12184

[CR69] Maggin, D. M., Swaminathan, H., Rogers, H. J., O’Keefe, B. V., Sugai, G., & Horner, R. H. (2011). A generalized least squares regression approach for computing effect sizes in single-case research Application examples. *Journal of School Psychology,**49*(3), 301–321. 10.1016/j.jsp.2011.03.004 21640246 10.1016/j.jsp.2011.03.004

[CR70] Manolov, R., & Moeyaert, M. (2025). Multilevel model selection applied to single-case experimental design data. *Journal of Behavioral Education*. Advance online publication. 10.1007/s10864-025-09593-9

[CR71] Manolov, R., Moeyaert, M., & Fingerhut, J. (2022). A priori justification for effect measures in single-case experimental designs. *Perspectives on Behavior Science,**45*(1), 156–189. 10.1007/s40614-021-00282-2 10.1007/s40614-021-00282-2 PMC8894525 35342872

[CR72] Manolov, R., & Onghena, P. (2018). Analyzing data from single-case alternating treatments designs. *Psychological Methods,**23*(3), 480–504. 10.1037/met0000133 28301199 10.1037/met0000133

[CR73] Manolov, R., & Onghena, P. (2022). Defining and assessing immediacy in single case experimental designs. *Journal of the Experimental Analysis of Behavior,**118*(3), 462–492. 10.1002/JEAB.799 36106573 10.1002/jeab.799 PMC9825864

[CR74] Manolov, R., Solanas, A., & Sierra, V. (2019). Extrapolating baseline trend in single-case data: Problems and tentative solutions. *Behavior Research Methods,**51*(6), 2847–2869. 10.3758/s13428-018-1165-x 30484219 10.3758/s13428-018-1165-x

[CR75] Manolov, R., Solanas, A., & Sierra, V. (2020). Changing criterion designs: Integrating methodological and data analysis recommendations. *The Journal of Experimental Education,**88*(2), 335–350. 10.1080/00220973.2018.1553838

[CR76] Manolov, R., & Tanious, R. (2022). Assessing consistency in single-case data features using modified Brinley plots. *Behavior Modification,**46*(3), 581–627. 10.1177/0145445520982969 33371723 10.1177/0145445520982969

[CR77] Manolov, R., & Tanious, R. (2025). Assessing nonoverlap in single-case data: Strengths, challenges, and recommendations. *Journal of Behavioral Education,**34*(4), 869–901. 10.1007/s10864-024-09552-w

[CR78] Manolov, R., Tanious, R., & Fernández-Castilla, B. (2022). A proposal for the assessment of replication of effects in single-case experimental designs. *Journal of Applied Behavior Analysis,**55*(3), 997–1024. 10.1002/jaba.923 35467023 10.1002/jaba.923 PMC9324994

[CR79] Manolov, R., & Vannest, K. (2023). A visual aid and objective rule encompassing the data features of visual analysis. *Behavior Modification,**47*(6), 1345–1376. 10.1177/0145445519854323 31165621 10.1177/0145445519854323

[CR80] Mensah, I., & Chen, L. T. (2025, March). ChatGPT as a support tool for visual analysis in singe-case design studies. In *Society for Information Technology & Teacher Education International Conference* (pp. 3403-3412). Association for the Advancement of Computing in Education (AACE).

[CR81] Michiels, B., & Onghena, P. (2019). Randomized single-case AB phase designs: Prospects and pitfalls. *Behavior Research Methods,**51*(6), 2454–2476. 10.3758/s13428-018-1084-x 30022457 10.3758/s13428-018-1084-x

[CR82] Moeyaert, M., Ferron, J., Beretvas, S., & den Van Noortgate, W. (2014). From a single-level analysis to a multilevel analysis of single-case experimental designs. *Journal of School Psychology,**52*(2), 191–211. 10.1016/j.jsp.2013.11.003 24606975 10.1016/j.jsp.2013.11.003

[CR83] Moeyaert, M., Ugille, M., Ferron, J., Beretvas, S. N., & den Van Noortgate, W. (2014). The influence of the design matrix on treatment effect estimates in the quantitative analyses of single-case experimental designs research. *Behavior Modification,**38*(5), 665–704. 10.1177/0145445514535243 24902590 10.1177/0145445514535243

[CR84] Moeyaert, M., & Yang, P. (2021). Assessing generalizability and variability of single-case design effect sizes using two-stage multilevel modeling including moderators. *Behaviormetrika,**48*(2), 207–229. 10.1007/s41237-021-00141-z

[CR85] Morley, S. (2018). *Single-case methods in clinical psychology: A practical guide*. Routledge.

[CR86] Natesan, P. (2019). Fitting Bayesian models for single-case experimental designs: A tutorial. *Methodology,**15*(4), 147–156. 10.1027/1614-2241/a000180

[CR87] Natesan Batley, P. (2023). Bayesian analysis of single case experimental design count data in trauma research: A tutorial. *Psychological Trauma: Theory, Research, Practice, and Policy,**5*(5), 829–837. 10.1037/tra0001357 10.1037/tra0001357 36455884

[CR88] Natesan, P., & Hedges, L. V. (2017). Bayesian unknown change-point models to investigate immediacy in single case designs. *Psychological Methods,**22*(4), 743–759. 10.1037/met0000134 28406673 10.1037/met0000134

[CR89] Ninci, J., Vannest, K. J., Willson, V., & Zhang, N. (2015). Interrater agreement between visual analysts of single-case data: A meta-analysis. *Behavior Modification,**39*(4), 510–541. 10.1177/0145445515581327 25878161 10.1177/0145445515581327

[CR90] Onghena, P., Tanious, R., De, T. K., & Michiels, B. (2019). Randomization tests for changing criterion designs. *Behaviour Research and Therapy,**117*, 18–27. 10.1016/j.brat.2019.01.005 30670306 10.1016/j.brat.2019.01.005

[CR91] Parker, R. I., & Vannest, K. J. (2009). An improved effect size for single-case research: Nonoverlap of all pairs. *Behavior Therapy,**40*(4), 357–367. 10.1016/j.beth.2008.10.006 19892081 10.1016/j.beth.2008.10.006

[CR92] Parker, R. I., Vannest, K. J., & Davis, J. L. (2011). Effect size in single-case research: A review of nine nonoverlap techniques. *Behavior Modification,**35*(4), 303–322. 10.1177/0145445511399147 21411481 10.1177/0145445511399147

[CR93] Parker, R. I., Vannest, K. J., Davis, J. L., & Sauber, S. B. (2011). Combining nonoverlap and trend for single-case research: Tau-U. *Behavior Therapy,**42*(2), 284–299. 10.1016/j.beth.2010.08.006 21496513 10.1016/j.beth.2010.08.006

[CR94] Perdices, M., Tate, R. L., & Rosenkoetter, U. (2023). An algorithm to evaluate methodological rigor and risk of bias in single-case studies. *Behavior Modification,**47*(6), 1482–1509. 10.1177/0145445519863035 31466459 10.1177/0145445519863035

[CR95] Pfadt, A., & Wheeler, D. J. (1995). Using statistical process control to make data-based clinical decisions. *Journal of Applied Behavior Analysis,**28*(3), 349–370. 10.1901/jaba.1995.28-349 7592154 10.1901/jaba.1995.28-349 PMC1279837

[CR96] Pustejovsky, J. E. (2018). Using response ratios for meta-analyzing single-case designs with behavioral outcomes. *Journal of School Psychology,**68*(Jun), 99–112. 10.1016/j.jsp.2018.02.003 29861034 10.1016/j.jsp.2018.02.003

[CR97] Pustejovsky, J. E., & Ferron, J. M. (2017). Research synthesis and meta-analysis of single-case designs. In J. M. Kauffman, D. P. Hallahan, & P. C. Pullen (Eds.), *Handbook of special education* (2nd ed., pp. 168–186). Routledge.

[CR98] Pustejovsky, J. E., Hedges, L. V., & Shadish, W. R. (2014). Design-comparable effect sizes in multiple baseline designs: A general modeling framework. *Journal of Educational and Behavioral Statistics,**39*(5), 368–393. 10.3102/1076998614547577

[CR99] Reichow, B., & Volkmar, F. R. (2010). Social skills interventions for individuals with autism: Evaluation for evidence-based practices within a best evidence synthesis framework. *Journal of Autism and Developmental Disorders,**40*(2), 149–166. 10.1007/s10803-009-0842-0 19655240 10.1007/s10803-009-0842-0

[CR100] Schlosser, R. W. (2009). *The role of single-subject experimental designs in evidence-based practice times*. (FOCUS: Technical Brief 22). National Center for the Dissemination of Disability Research (NCDDR). Retrieved May 24, 2018, from http://ktdrr.org/ktlibrary/articles_pubs/ncddrwork/focus/focus22/Focus22.pdf

[CR101] Shadish, W. R., Hedges, L. V., & Pustejovsky, J. E. (2014). Analysis and meta-analysis of single-case designs with a standardized mean difference statistic: A primer and applications. *Journal of School Psychology,**52*(2), 123–147. 10.1016/j.jsp.2013.11.005 24606972 10.1016/j.jsp.2013.11.005

[CR102] Shadish, W. R., Kyse, E. N., & Rindskopf, D. M. (2013). Analyzing data from single-case designs using multilevel models: New applications and some agenda items for future research. *Psychological Methods,**18*(3), 385–405. 10.1037/a0032964 23834421 10.1037/a0032964

[CR103] Shadish, W. R., & Sullivan, K. J. (2011). Characteristics of single-case designs used to assess intervention effects in 2008. *Behavior Research Methods,**43*(4), 971–980. 10.3758/s13428-011-0111-y 21656107 10.3758/s13428-011-0111-y

[CR104] Slocum, T. A., Joslyn, P. R., Nichols, B., & Pinkelman, S. E. (2022). Revisiting an analysis of threats to internal validity in multiple baseline designs. *Perspectives on Behavior Science,**45*(3), 681–694. 10.1007/s40614-022-00351-0 36249172 10.1007/s40614-022-00351-0 PMC9458797

[CR105] Snodgrass, M., Cook, B. G., & Cook, L. (2023). Considering social validity in special education research. *Learning Disabilities Research & Practice,**38*(4), 311–319. 10.1111/ldrp.12326

[CR106] Speelman, C. P., & McGann, M. (2020). Statements about the pervasiveness of behaviour require data about the pervasiveness of behavior. *Frontiers in Psychology,**11*, Article 594675. 10.3389/fpsyg.2020.594675 33329258 10.3389/fpsyg.2020.594675 PMC7711086

[CR107] Steenbeek, D., Ketelaar, M., Lindeman, E., Galama, K., & Gorter, J. W. (2010). Interrater reliability of goal attainment scaling in rehabilitation of children with cerebral palsy. *Archives of Physical Medicine and Rehabilitation,**91*(3), 429–435. 10.1016/j.apmr.2009.10.013 20298835 10.1016/j.apmr.2009.10.013

[CR108] Swan, D. M., & Pustejovsky, J. E. (2018). A gradual effects model for single-case designs. *Multivariate Behavioral Research,**53*(4), 574–593. 10.1080/00273171.2018.1466681 29757002 10.1080/00273171.2018.1466681

[CR109] Tanious, R., De, T. K., Michiels, B., den Van Noortgate, W., & Onghena, P. (2020). Assessing consistency in single-case A-B-A-B phase designs. *Behavior Modification,**44*(4), 518–551. 10.1177/0145445519837726 30931585 10.1177/0145445519837726

[CR110] Tanious, R., & Manolov, R. (2022). Violin plots as visual tools in the meta-analysis of single-case experimental designs. *Methodology,**18*(3), 221–238. 10.5964/meth.9209

[CR111] Tanious, R., & Manolov, R. (2025). Proposals for incorporating the reversal in single-case ABAB designs data analysis. *The Journal of Experimental Education,**93*(4), 787–801. 10.1080/00220973.2024.2382485

[CR112] Tanious, R., Manolov, R., & Onghena, P. (2021). The assessment of consistency in single-case experiments: Beyond A-B-A-B designs. *Behavior Modification,**45*(4), 560–580. 10.1177/0145445519882889 31619052 10.1177/0145445519882889

[CR113] Tanious, R., Manolov, R., Onghena, P., & Vlaeyen, J. W. S. (2024). Single-case experimental designs: The importance of randomization and replication. *Nature Reviews Methods Primers,**4*(1), Article 27. 10.1038/s43586-024-00312-8

[CR114] Tanious, R., & Onghena, P. (2021). A systematic review of applied single-case research published between 2016 and 2018: Study designs, randomization, data aspects, and data analysis. *Behavior Research Methods,**53*(4), 1371–1384. 10.3758/s13428-020-01502-4 33104956 10.3758/s13428-020-01502-4

[CR115] Tarlow, K. (2017). An improved rank correlation effect size statistic for single-case designs: Baseline corrected Tau. *Behavior Modification,**41*(4), 427–467. 10.1177/0145445516676750 27831527 10.1177/0145445516676750

[CR116] Tate, R. L., & Perdices, M. (2019). *Single-case experimental designs for clinical research and neurorehabilitation settings: Planning, conduct, analysis, and reporting*. Routledge.

[CR117] Tate, R. L., Perdices, M., Rosenkoetter, U., Shadish, W., Vohra, S., Barlow, D. H., . . . Wilson, B. (2016). The Single-Case Reporting Guideline In BEhavioural Interventions (SCRIBE) 2016 statement. *Archives of Scientific Psychology,**4*(1), 1–9. 10.1037/arc0000026 10.1016/j.jsp.2016.04.001 27268573

[CR118] Tate, R. L., Perdices, M., Rosenkoetter, U., Wakim, D., Godbee, K., Togher, L., & McDonald, S. (2013). Revision of a method quality rating scale for single-case experimental designs and n-of-1 trials: The 15-item Risk of Bias in N-of-1 Trials (RoBiNT) Scale. *Neuropsychological Rehabilitation,**23*(5), 619–638. 10.1080/09602011.2013.824383 24050810 10.1080/09602011.2013.824383

[CR119] Tincani, M., Gilroy, S. P., & Dowdy, A. (2024). Extensions of open science for applied behavior analysis: Preregistration for single-case experimental designs. *Journal of Applied Behavior Analysis,**57*(4), 808–820. 10.1002/jaba.2909 39140415 10.1002/jaba.2909

[CR120] Tincani, M., & Travers, J. (2018). Publishing single-case research design studies that do not demonstrate experimental control. *Remedial and Special Education,**39*(2), 118–128. 10.1177/0741932517697447

[CR121] Tincani, M., Travers, J., Dowdy, A., Slocum, T. A., & Deitrich, R. (2025). Questionable and improved research practices in single-case experimental design: Initial investigation and findings. *Perspectives on Behavior Science,**48*(2), 447–473. 10.1007/s40614-025-00441-9 40520582 10.1007/s40614-025-00441-9 PMC12162424

[CR122] Turgeon, S., & Lanovaz, M. J. (2020). Tutorial: Applying machine learning in behavioral research. *Perspectives on Behavior Science,**43*(4), 697–723. 10.1007/s40614-020-00270-y 33381685 10.1007/s40614-020-00270-y PMC7724016

[CR123] Valentine, J. C., Tanner-Smith, E. E., & Pustejovsky, J. E. (2016). Between-case standardized mean difference effect sizes for single-case designs: A primer and tutorial using the scdhlm web application. *The Campbell Collaboration*. 10.4073/cmdp.2016.1

[CR124] den Van Noortgate, W., & Onghena, P. (2003). Combining single-case experimental studies using hierarchical linear models. *School Psychology Quarterly,**18*(3), 325–346. 10.1521/scpq.18.3.325.22577

[CR125] den Van Noortgate, W., & Onghena, P. (2003). Hierarchical linear models for the quantitative integration of effect sizes in single-case research. *Behavior Research Methods,**35*(1), 1–10. 10.3758/BF03195492 10.3758/bf03195492 12723775

[CR126] Vannest, K. J., Parker, R. I., Davis, J. L., Soares, D. A., & Smith, S. L. (2012). The Theil–Sen slope for high-stakes decisions from progress monitoring. *Behavioral Disorders,**37*(4), 271–280. 10.1177/019874291203700406

[CR127] Verboon, P., & Peters, G. J. (2020). Applying the generalized logistic model in single case designs: Modeling treatment-induced shifts. *Behavior Modification,**44*(1), 27–48. 10.1177/0145445518791255 30079759 10.1177/0145445518791255 PMC6873223

[CR128] What Works Clearinghouse. (2022). Procedures and Standards Handbook, Version 5.0. U.S. Department of Education, Institute of Education Sciences. Retrieved from https://ies.ed.gov/ncee/wwc/Docs/referenceresources/Final_WWC-HandbookVer5.0-0-508.pdf

[CR129] Wilbert, J., Bosch, J., & Lüke, T. (2021). Validity and judgment bias in visual analysis of single-case data. *International Journal for Research in Learning Disabilities,**5*(1), 13–24. 10.28987/ijrld.5.1.13

[CR130] Wolery, M., Busick, M., Reichow, B., & Barton, E. E. (2010). Comparison of overlap methods for quantitatively synthesizing single-subject data. *The Journal of Special Education,**44*(1), 18–29. 10.1177/0022466908328009

[CR131] Wolfe, K., Barton, E. E., & Meadan, H. (2019). Systematic protocols for the visual analysis of single-case research data. *Behavior Analysis in Practice,**12*(2), 491–502. 10.1007/s40617-019-00336-7 31976257 10.1007/s40617-019-00336-7 PMC6745757

[CR132] Wolfe, K., McCammon, M. N., LeJeune, L. M., Check, A. R., & Slocum, T. A. (2024). A review of visual analysis reporting procedures in the functional communication training literature. *School Psychology,**39*(6), 548–556. 10.1037/spq0000660 39298212 10.1037/spq0000660

